# Libertellenone T, a Novel Compound Isolated from Endolichenic Fungus, Induces G2/M Phase Arrest, Apoptosis, and Autophagy by Activating the ROS/JNK Pathway in Colorectal Cancer Cells

**DOI:** 10.3390/cancers15020489

**Published:** 2023-01-12

**Authors:** Chathurika D. B. Gamage, Jeong-Hyeon Kim, Yi Yang, İsa Taş, So-Yeon Park, Rui Zhou, Sultan Pulat, Mücahit Varlı, Jae-Seoun Hur, Sang-Jip Nam, Hangun Kim

**Affiliations:** 1 College of Pharmacy and Research Institute of Life and Pharmaceutical Sciences, Sunchon National University, 255 Jungang-ro, Sunchon, Jeonnam 57922, Republic of Korea; 2 Department of Chemistry and Nanoscience, Ewha Womans University, Seoul 03760, Republic of Korea; 3 Korean Lichen Research Institute, Sunchon National University, 255 Jungang-ro, Sunchon, Jeonnam 57922, Republic of Korea

**Keywords:** CRC, Libertellenone T, G2/M phase arrest, apoptosis, autophagy, ROS/JNK signaling

## Abstract

**Simple Summary:**

Libertellenone T (**B**) is a natural product derived from the secondary metabolites of the endolichenic fungus, *Pseudoplectania* sp. In this study, we investigated the underlying molecular mechanisms that induce the apoptotic cell death of the colorectal cancer cell line, Caco2, in response to **B**. Our findings demonstrate that **B** induces Caco2 cell apoptosis via G2/M phase arrest and activation of ROS/JNK signaling. Moreover, **B** exhibited excellent synergistic effects when combined with the known and novel anticancer agents 5-FU and compound D, respectively. In light of this investigation, we propose that **B** is a promising potential chemotherapeutic agent against colorectal cancer.

**Abstract:**

Colorectal cancer (CRC) is the third most deadly type of cancer in the world and continuous investigations are required to discover novel therapeutics for CRC. Induction of apoptosis is one of the promising strategies to inhibit cancers. Here, we have identified a novel compound, Libertellenone T (**B**), isolated from crude extracts of the endolichenic fungus from *Pseudoplectania* sp. (EL000327) and investigated the mechanism of action. CRC cells treated by **B** were subjected to apoptosis detection assays, immunofluorescence imaging, and molecular analyses such as immunoblotting and QRT-PCR. Our findings revealed that **B** induced CRC cell death via multiple mechanisms including G2/M phase arrest caused by microtubule stabilization and caspase-dependent apoptosis. Further studies revealed that **B** induced the generation of reactive oxygen species (ROS) attributed to activating the JNK signaling pathway by which apoptosis and autophagy was induced in Caco2 cells. Moreover, **B** exhibited good synergistic effects when combined with the well-known anticancer drug, 5-FU, and another cytotoxic novel compound D, which was isolated from the same crude extract of EL000327. Overall, Libertellenone T induces G2/M phase arrest, apoptosis, and autophagy via activating the ROS/JNK pathway in CRC. Thus, **B** may be a potential anticancer therapeutic against CRC that is suitable for clinical applications.

## 1. Introduction

An abnormal cell growth that occurs in the colon or rectum is simply recognized as colorectal cancer (CRC). A noncancerous cell growth, known as a polyp, can develop in the mucosal layer of the colon or rectum, and may develop into CRC. Less than 10% of polyps have a high potential to progress into invasive cancer over 10–20 years. CRC occurs predominantly in adults aged 50 and older [1]. In 2020, CRC was recognized as the third most common cancer type worldwide and the second most common cause of cancer death. Geographically, the occurrence of CRC is highest in Asian regions. The International Agency for Research on Cancer (IARC) estimated that the global burden of CRC will increase by 56%, and CRC related mortality will increase by 69% between 2020 and 2040 [2]. Therefore, a strong research focus on the discovery of novel anticancer therapeutics is urgently required.

Many scientists have focused their attention on the development of innovative drugs derived from natural sources such as plants, lichens, and micro-organisms over a few decades. A lichen is a symbiotic living form associated with a fungus (mycobiont) and a cyanobacterium or green alga (the photobiont), or both [2]. Some endolichenic fungi (ELF) reside inside the lichen thalli and produce bioactive secondary metabolites with medicinal and economic potential. These secondary metabolites show cytotoxic, antioxidant, antifungal, and antibacterial bioactivities, which are crucial in drug development in the pharmaceutical industry [3,4]. Therefore, the ability of ELF to produce unique secondary metabolites with anti-cancer properties provides a novel opportunity to identify effective cancer therapeutics [5,6,7]. Libertellenone T (**B**) is a novel cytotoxic compound isolated from secondary metabolites extracted from the endolichenic fungus, EL000327, from *Pseudoplectania* sp. found in the lichen *Graphis*, collected from Hallasan in Jeju Island, South Korea in 2009. 

Many chemotherapeutics exert cytotoxicity and trigger cancer cell death via inducing apoptosis in cells. Apoptosis, or programmed cell death, is a cell suicide process that activates when their continuous survival is blocked [8]. Morphological and biochemical changes such as chromatin condensation, nuclear fragmentation, cell shrinkage, membrane blebbing, DNA, protein breakdown, and caspase activation can be observed in apoptotic cells. Cancer cells evade natural cell death as a result of genetic mutations acquired during transformation from normal cells to malignant cells. Disruption of the balance between pro-apoptotic and anti-apoptotic proteins, reduction in caspase function, and impairment of death receptor signaling all cause apoptosis resistance in cancer cells [9]. Therefore, restoration of these functions by therapeutics can successfully prevent cancer progression. Furthermore, alterations that occur in cell cycle regulators at check points cause abnormal cell proliferation in cancers [10]. Thus, the induction of G2/M phase arrest is another target of many anti-cancer agents. Controlling cell progression through the cell cycle by regulating related proteins or disrupting tubulin organization eventually leads cells to G2/M phase arrest and subsequent cell death [11,12].

Autophagy (macroautophagy) mainly involves the formation of an autophagosome by engulfing damaged organelles, fusion with lysosome, and degradation of cellular debris by lysosomal hydrolase [13,14]. Autophagy plays a paradoxical role in cancer progression. In the early stage of many cancers, autophagy acts as a tumor suppressor by protecting cell homeostasis. Conversely, autophagy promotes tumor growth in more advanced stages of cancer by increasing stress tolerance [15]. Many anticancer therapeutics are known to activate autophagy concurrently with apoptosis. This activation of autophagy can either promote or suppress cancer cell survival. Therefore, the role of autophagy is crucial in anticancer drug development [16]. Furthermore, the relationship between autophagy and apoptosis is unclear and yet to be investigated. 

Reactive oxygen species (ROS) are highly reactive and short-lived small molecules in the form of free radicals [17]. ROS support many physiological functions in cells under optimum conditions. However, the over production of ROS can have a deleterious effect on cells and trigger oxidative stress. The generation of ROS can be triggered by both endogenous and exogenous sources such as mitochondrial transport chain leakage, high metabolic rate, environmental pollutants, radiation, and drugs [18]. ROS are responsible for activating many cellular signaling pathways that lead to cell survival or cell death including autophagy, apoptosis, and necrosis. c-Jun N-terminal kinase (JNK) signaling is activated in response to ER stress or activation of the mitochondrial pathway of apoptosis by ROS [19].

In this study, we investigated the possible mechanisms underlying the impact of **B** on CRC cells. We selected four CRC cell lines, HCT116, DLD1, HT29, and Caco2, harboring different genetic mutations and status of microsatellite instability, to evaluate the effect of **B** on them. HCT116 is a microsatellite instable (MSI) cell line with KRAS and PIK3CA mutations. DLD1 is the MSI cell line, harboring KRAS, PIK3CA, and TP53 mutations. HT29 is microsatellite stable (MSS), and BRAF and PIK3CA are muted cell lines, while Caco2 is MSS and the wild type of KRAS PIK3CA, TP53 [20]. Furthermore, we demonstrated that **B** induced mitotic arrest, apoptosis, and autophagy via activating the ROS/JNK signaling pathway. In addition, we found that **B**-induced autophagy promotes cell survival. Furthermore, **B** exhibited synergy not only with the known anticancer drug *5-Fluorouracil* (5-FU), but also with a novel compound D. Collectively, our data suggest that **B** is a potential candidate as an anticancer therapeutic with clinical application.

## 2. Materials and Methods

### 2.1. Fungal Strain

Lichen specimens of *Graphis* were collected from Hallasan in Jeju Island, South Korea in 2009. Voucher specimen was deposited in the Korean Lichen Research Institute, Sunchon National University, Korea. The endolichenic fungus EL000327 was isolated with the surface sterilization method [21].

### 2.2. ITS Sequencing

EL000327 was cultured for 2–3 weeks on potato dextrose agar (PDA) medium at 25 °C. The total DNA was extracted following the manufacturer’s instructions from EL000327 using the DNeasy Plant Mini Kit (Qiagen, Hilden, Germany). Universal primers ITS1F (5’-CTTGGTCATTTAGAGGAAGTAA-3’) [22] and LR5 (5’-ATCCTGAGGGAAACTTC-3’) [23] were amplified with the internal transcribed spacer (ITS) region of the rDNA gene of EL000327 and ITS sequencing was performed as described [5] (Table S1).

### 2.3. Preparation of Secondary Metabolite Extract of EL000327

EL000327 was cultured on potato dextrose agar (PDA) medium at 25 °C for approximately 2–3 weeks until visible colonies were evident. ELF mycelia grown on agar were cut and inoculated into 200 mL potato dextrose broth (PDB) in 500 mL Erlenmeyer flasks (3 L) and incubated at 25 °C in a shaking incubator at 150 rpm for approximately 3–4 weeks. Then, 200 mL ethyl acetate (EA) was added to each flask, and the flask was shaken for approximately 2 h. Each culture was then filtered to separate the filtrate and mycelia. The filtrate was separated into water- and EA-soluble layers by allowing the filtrate to stand in a separating funnel. A total of 5.8 g of crude extracts of EL000327 was obtained by evaporating EA to dryness under a vacuum using a rotary evaporator. The crude extract was dissolved in 100% DMSO for use in experiments.

### 2.4. Isolation, Purification, and Identification of Chemical Structure of Compound **B**

The crude extract (5.8 g) was subjected to open column chromatography purification on a RP C18 flash column by the step gradient elution of methanol/H_2_O from 20% to 100% of methanol, subsequently, to afford eight fractions (labeled 327-F1 ~ 327-F8). Fraction 327-F2 (360 mg) (H_2_O:MeOH = 60:40) was purified by reversed-phase HPLC (Phenomenex Luna C-18 (2), 250 × 100 mm, 2.0 mL/min, 5 μm, 100 Å, UV = 254 nm) (Figure S8) using an isocratic solvent system with 47% acetonitrile in water to yield 7β-9α-dihydroxy-1,8(14),15-pimaratrien-3,11-dione (B, 95 mg, purity: 96.7%) as pink oil. ^1^H NMR (400 MHz, CD_3_OD) *δ*: 7.25 (d, *J* = 10.4 Hz, 1H), 6.02 (d, *J* = 2.2 Hz, 1H), 5.93 (d, *J* = 10.4 Hz, 1H), 5.74 (dd, *J* = 17.2, 10.4 Hz, 1H), 4.99 (dd, *J* = 17.3, 0.9 Hz, 1H), 4.95 (dd, *J* = 10.4, 0.9 Hz, 1H), 4.40 (m, 1H), 1.29 (s, 3H), 1.20 (s, 3H), 1.18 (s, 3H); ^13^C NMR (100 MHz, CD_3_OD) *δ*: 212.1, 206.3, 159.5, 145.4, 141.5, 130.9, 128.8, 69.6, 54.0, 45.7, 45.3, 43.2, 42.9, 33.2, 28.5, 28.1, 22.8, 20.8. HR-FAB-MS *m*/*z* [M+H]^+^ 331.1904 (calcd. for C_20_H_27_O_4_, 331.1909) (Figures S6 and S7).

### 2.5. Cell Culture

Human CRC cell lines HT29, HCT116, DLD1, Caco2, colon stemness cancer cell line; CSC221, human gastric cancer cell lines; AGS, TMK1, human prostate cancer cell line; RV1, human lung cancer cell line; A549, mouse colon cancer cell line; CT26 and canine kidney epithelial cell line; and MDCK were purchased from the Korean Cell Line Bank (Seoul, Korea). Cells were cultured in DMEM or RPMI culture medium (GenDEPOT, Katy, TX, USA) supplemented with 10% fetal bovine serum (FBS) (GenDEPOT, Katy, TX, USA) and 1% penicillin–streptomycin solution, and incubated in a humidified atmosphere at 37 °C in 5% CO_2_.

### 2.6. Cell Viability Assay

The viability of the cells was determined using the 3-(4,5-dimethylthiazol-2-yl)-2,5-diphenyltetrazolium bromide (MTT) colorimetric assay (Sigma-Aldrich, St. Louis, MO, USA). The 2 × 10^4^–4 × 10^4^ cells/mL were seeded in 96-well plates. After the attachment, cells were treated with different concentrations of **B**, EL000327, D, and 5-FU for 48 h in the presence or absence of various inhibitors (Z-VAD-FMK (10 µM) (R&D System, Inc, McKinley Place N.E, MN, USA), 3-MA (1mM), CQ (10 µM), NAC (5 mM) (Sigma-Aldrich, St. Louis, MO, USA), and SP600125 (10 µM) (Cell Signaling Technology, MA, USA). Cells treated with 0.01% of DMSO were used as the control. Next, 15 µL of the MTT reagent was added to each well and incubated for 4 h at 37 °C. The medium was aspirated completely and 150 µL of DMSO (Sigma-Aldrich, St. Louis, MO, USA) was added to the cells before the absorbance was measured at 540 nm by a microplate reader (Bio Tek Instruments, Winooskim, VT, USA) using Gen 5 (2.03.1) software. SPSS statistical software 23 was used for the IC_50_ calculation. Synergic effects of **B** with D or 5-FU were assessed by compuSyn software.

### 2.7. Cell Cycle Analysis by Flow Cytometry

Caco2, HCT116, DLD1, and HT29 cells were seeded in 6-well plates at the density of 1.5–2 × 10^5^ cells/well, incubated overnight, and treated with 0.01% of DMSO, various concentrations of **B**, and 60 µg/mL of EL000327 for 24 h, 48 h, or 72 h. Cells were harvested and washed with FACS washing buffer, incubated with trypsin solution, followed by RNase A for 10 min at room temperature. Cells were centrifuged and pellets were collected and stained with 100 mL of 4 mg/mL PI (Sigma-Aldrich, St. Louis, MO, USA) for 2 h in the dark at 4 °C. Cell cycle analysis was performed on a CytoFLEX instrument (Beckman Coulter Life Sciences, Indianapolis, IN, USA).

### 2.8. Western Blotting

Caco2 cells were cultured in 6-well plates at the density of 2 × 10^5^ cells/well overnight and treated with 0.01% of DMSO, different concentrations of **B**, and 60 µg/mL of EL000327 for 24 h or 48 h in the presence or absence of various inhibitors (Z-VAD-FMK (10 µM), 3-MA (1 mM), CQ (10 µM), NAC (5 mM), and SP600125 (10 µM). Cells were harvested and lysed, and the protein concentrations were determined by the BCA protein assay following the manufacturer’s instructions. Then, 25 or 50 µg of the total extract was separated by SDS-PAGE (12%) and transferred to a blotting membrane at 1.2 A for 6 h. Membranes were blocked with 5% of skim milk for 1 h followed by incubation with various primary antibodies (Cyclin B1, D1, p-Cdc2, BAX, Bcl-XL, PARP, caspase-3, Beclin 1, P-62, LC3B I/II, p-JNK, JNK, p-Akt, Akt, NF-κB, Actin purchased from Cell Signaling Technology, MA, USA) for 2 h at room temperature (RT). Blots were washed and incubated with horseradish peroxidase-conjugated secondary antibodies (Thermo Fisher Scientific, Waltham, MA, USA) for 30 to 60 min at RT. Specific antibody binding was detected under chemiluminescence imaging (iBright FL1000 Imaging System, Thermo Fisher Sciences, biomolecular imager, Amersham ImageQuantTM 800 Western Blot Imaging System) and measured by Multi Gauge 3.0. software. Relative density was calculated against the density of the actin bands.

### 2.9. Tubulin Polymerization Assay

The effect of **B** on tubulin organization was detected using the Tubulin Polymerization Assay Kit (Cytoskeleton, Inc., Denver, CO, USA) according to the manufacturer’s instructions. In brief, tubulin proteins (>99% pure) were suspended at a final concentration of 3.0 mg/mL in ice-cold TP, and the tubulin solution was incubated at 37 °C with a general tubulin buffer with or without **B** (3.3, 20, 60 µg/mL). Paclitaxel and vinblastine (10 µM) were used as positive controls for the stabilization or destabilization of the microtubules, respectively. Tubulin polymerization was measured by continuously monitoring the change in turbidity at 340 nm by a microplate reader using Gen 5 (2.03.1) software.

### 2.10. Quantitative Real-Time PCR

Total RNA of 0.01% of DMSO, **B** (20, 60 µg/mL), and EL000327 (60 µg/mL) treated Caco2 cells were extracted using RNAiso Plus (TaKaRa, Kusatsu, Shiga, Japan) according to the manufacturer’s instructions. cDNA was reverse transcribed from 3 µg of total RNA of each treated group using the M-MLV Reverse Transcriptase Kit (Invitrogen, Carlsbad, CA, USA). mRNA levels of stathmin and MAP4 was measured using stathmin (forward) 5-GGTGGCGGCAGGACTTTCCTTATCCCAGTTGATT-3 and (reverse) 5- TTCTCGTGCTCTCGTTTCTCAGCCAGCTGCTTC-3; MAP4 (forward) 5-CCCTTTCTGAGGTAGCGTGCCTTGTGGAGGT -3 and (reverse) 5-CTGGCTCCCTCATGTTCTTGGCACAGCAGA-3 primers and SYBR green (Enzynomics). qRT-PCR reaction and analysis were performed using CFX (Bio-Rad, Hercules, CA, USA).

### 2.11. Immunofluorescence (IF) Imaging

Caco2 cells were cultured on cover slips at the density of 1 × 10^5^ in a 12-well plate. After the adherence, cells were treated with 0.01% of DMSO, different concentrations of **B**, paclitaxel (100 nM), vinblastine (50 nM), and deoxyphodophyllotoxin (DPT) (25 nM) for 24 h. Cells were washed with phosphate-buffered saline (PBS) three times, followed by fixation with 4% paraformaldehyde in PBS for 10 min, permeabilization with 0.1% Triton™ X-100 for 10 min at RT, and blocking with 1% BSA in PBS for 1 h at RT. The cells were labeled with alpha tubulin (B-5-1-2) Alexa Fluor 488 Mouse Monoclonal Antibody, at 2 µg/mL in 0.1% BSA, and incubated for 3 h at RT. Cells were washed three times with PBS for 5 min after every step. Then, the cells were blocked again with blocking solution containing 1% BSA for 30–45 min at RT. Cells were stained again with fluorescent phalloidin staining solution and incubated for 30–60 min at RT. After washing with PBS, cover slips were mounted on glass slides with prolong gold with DAPI and left overnight at RT. Images were taken using a K1-Fluo Confocal Laser Scanning Microscope (Nanoscope Systems, Daejeon, Republic of Korea).

### 2.12. Hoechst Staining

Caco2, HCT116, DLD1, and HT29 cells were seeded in a 12-well plate containing cover slips at a density of 1 × 10^5^ cells/well. After overnight incubation, cells were treated with 0.01% of DMSO, **B** (20, 60 µg/mL), and EL000327 (60 µg/mL) for 12 or 24 h. Cells were washed with PBS, followed by fixation with 4% paraformaldehyde for 15 min. After washing again with PBS, cells were permeabilized in 0.1% Triton X-100 (Sigma-Aldrich) for 30 min, and stained with Hoechst 33258 (Sigma-Aldrich) for 1 h in the dark at room temperature. Cells were assessed by Nikon Eclipse 400 fluorescence microscope (Nikon Instech Co. Ltd., Kawasaki, Japan) to identify the morphological changers in the nuclei.

### 2.13. IncuCyte™ Caspase-3/7 and Annexin v Apoptosis Assay

Caco2 cells were seeded in a 96-well plate at a density of 2.5 × 10^3^ cells/well. Cells were grown overnight to 25–30% confluence at the start of the assay. Media were supplemented with 5 μM of Caspase-3/7 (green) reagent (4440, Essen Bioscience, Morgan Rd, Ann Arbor, MI, USA) or Annexin V (red) reagent (4641, Essen Bioscience, Morgan Rd, Ann Arbor, MI, USA) diluted to 1:200 and added to the cells treated with 0.01% of DMSO, different concentrations of **B**, and EL000327 for 48 h in the presence or absence of Z-VAD-FMK (10 µM). The apoptosis of cells was determined by fluorescence scanning performed every 2 h for 48 h by the IncuCyte Zoom^®^ instrument with a 10× objective and analyzed with the Standard Scan Type.

### 2.14. Apoptosis Analysis by Flow Cytometry

Caco2, HCT116, DLD1, and HT29 cells were cultured in a 6-well plate at the density of 2 × 10^5^ cells/well until adherence. Cells were treated with 0.01% of DMSO, different concentrations of **B**, and EL000327 for 48 h in the presence or absence of Z-VAD-FMK (10 µM). Cells were harvested and washed with PBS, resuspended in 100 µL of 1x binding buffer followed by staining with 5 µL of 50 µg/mL propidium iodide (PI; BD Biosciences, San Jose, CA, USA) and 3 µL of Annexin V–FITC (BD, Biosciences, San Jose, CA, USA), for 30 min in the dark. Death cells were detected by flow cytometry on a CytoFLEX instrument (Beckman Coulter Life Sciences, Indianapolis, IN, USA).

### 2.15. Measurement of ROS Generation

Caco2 cells were seeded in a 6-well plate at the density of 2 × 10^5^ cells/well overnight and treated with 0.01% of DMSO, different concentrations of **B**, and EL000327 for 12 h in the presence or absence of NAC (5 mM). Cells were incubated with DCFH-DA (10 µM) in DMEM medium without FBS for 30 min at 37 °C and washed three times with DMEM. ROS generation was determined by fluorescence microscopy (K1-Fluo Confocal Laser Scanning Microscope, Nanoscope Systems, Daejeon, Republic of Korea) and flow cytometry (CytoFLEX; Beckman Coulter Life Sciences, Indianapolis, IN, USA) using peroxide-sensitive fluorescence probe DCFH-DA.

### 2.16. Statistical Analysis

All experiments were performed at least three times. Data are expressed as means ± standard deviation (SD). All statistical analyses were performed using Sigma Plot version 12.5. The Student’s *t*-test was used to compare the statistical significance between two groups. Unless indicated otherwise, a *p*-value < 0.05 was considered significant.

## 3. Results

### 3.1. The Novel Compound, **B**, Isolated from Crude Extract, EL000327, Exerts Cytotoxicity on the CRC Cells

**Figure 1:**
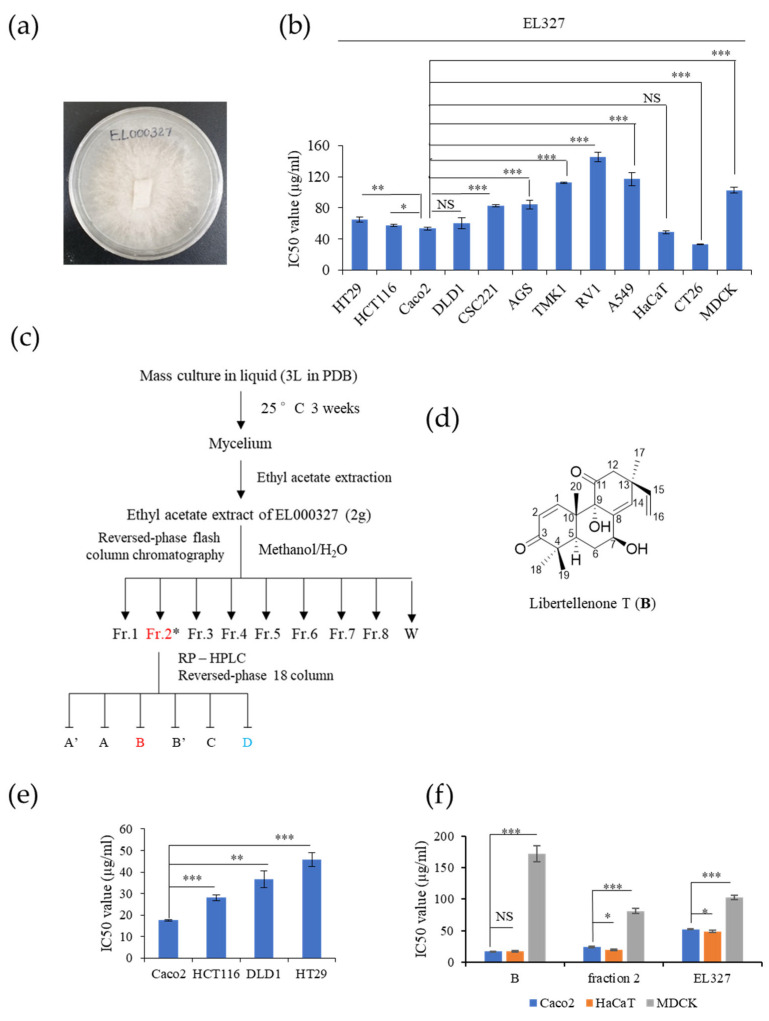
Compound **B** isolated from the crude extract of EL000327 exhibited cytotoxicity toward the human CRC cell line, Caco2. (**a**) Image of the endolichenic fungus, EL000327, belonging to *Pseudoplectania* sp. isolated from the lichen Graphis. (**b**) IC_50_ values of EL000327 in HT29, HCT116, Caco2, DLD1, CSC221, TMK1, RV1, A549, HaCaT, CT26, and MDCK cells. (**c**) Schematic representation of the process for the purification of compound **B** from the crude extract, EL000327. (**d**) Chemical structure of the novel compound, **B**. (**e**) IC_50_ values of human CRC cells Caco2, HCT116, DLD1, and HT29 after treatment with **B** for 48 h. (**f**) Comparison of IC_50_ values of the CRC cells Caco2 and non-cancer cell lines HaCaT and MDCK treated with single compound **B**, fraction 2, or crude extract, EL000327 for 48 h. Results are representative of three independent experiments. Data represent the mean ± S.D. * *p* < 0.05, ** *p* < 0.01, *** *p* < 0.001, NS: no significant difference (*p* > 0.05) compared with the Caco2 cells.

The endolichenic fungus EL000327 (Figure 1a) was isolated from lichen specimen Graphis, collected from Hallasan in Jeju Island, South Korea in 2009 using the surface sterilization method. EL000327 was identified as a *Pseudoplectania* sp. according to a BLAST search of the GeneBank Database (Table S1). The cytotoxicity of the crude extract of EL000327 was tested against several human cancer cell lines: HT29, HCT116, Caco2, DLD1, CSC221, AGS, TMK1, RV1, A549, a mouse colon cancer cell line CT26, and non-cancerous cell lines HaCaT and MDCK. Among the cancer cell lines, EL000327 showed the highest cytotoxicity toward the human CRC cell line, Caco2 (IC_50_ = 52.2 μg/mL) and the mouse colon cancer cell line, CT26 (IC_50_ = 33.12 μg/mL). Cytotoxicity on the spontaneously transformed human keratinocyte cell (HaCaT) was similar to that of the Caco2 cells (IC_50_ = 48.7 μg/mL) (Figure 1b). The extract of EL000327 was subjected to a purification process to isolate and identify active compounds from the crude extract. First, the extract of EL000327 was separated into seven fractions, and Fr.2 (IC_50_ = 24.35 μg/mL) was identified as the fraction with the strongest cytotoxicity against Caco2 cells. In a further purification of Fr.2, six purified compounds (A’, A, **B**, B’, C, D) were isolated, as indicated in Figure 1c.

Compound **B** was isolated as a pink oil, and its molecular formula was deduced as C_20_H_26_O_4_, based on the HR-FAB-MS data. The chemical structure of **B** was determined to be a novel compound 7β-9α-dihydroxy-1,8(14),15-pimaratrien-3,11-dione (Libertellenone T), based on intensive interpretation of MS, UV, and NMR spectroscopic data (Figure 1d). Furthermore, the stereo-configurations of compound **B** were determined by comparing the NMR spectroscopic data and the values of optical rotation with the literature [24].

The cytotoxicity of purified compound **B** on Caco2, HCT116, DLD1, HT29, HaCaT, and MDCK cell lines was evaluated. **B** was toxic to the Caco2 (IC_50_ = 17.5 μg/mL) cell line at much lower doses than the HCT116 (IC_50_ = 28 μg/mL), DLD1 (IC_50_ = 36.6 μg/mL), and HT29 (IC_50_ = 28 μg/mL) cell lines and the non-cancer cell lines, HaCaT (IC_50_ = 17.6 μg/mL) and MDCK (IC_50_ = 171.8 μg/mL) (Figure 1e). Furthermore, for MDCK cells, the IC_50_ of **B** was significantly higher than those of Fr.2 (IC_50_ = 81.35 μg/mL) or the crude extract EL000327 (IC_50_ = 102.5 μg/mL) (Figure 1f). Thus, **B** was identified as a novel chemical compound, which exerted the highest cytotoxicity toward Caco2 among the tested human CRC cell lines, and was suitable for further investigation of its mechanism of action.

### 3.2. **B** Induces G2/M Phase Arrest in CRC Cells as a Result of Microtubule Stabilization

**Figure 2:**
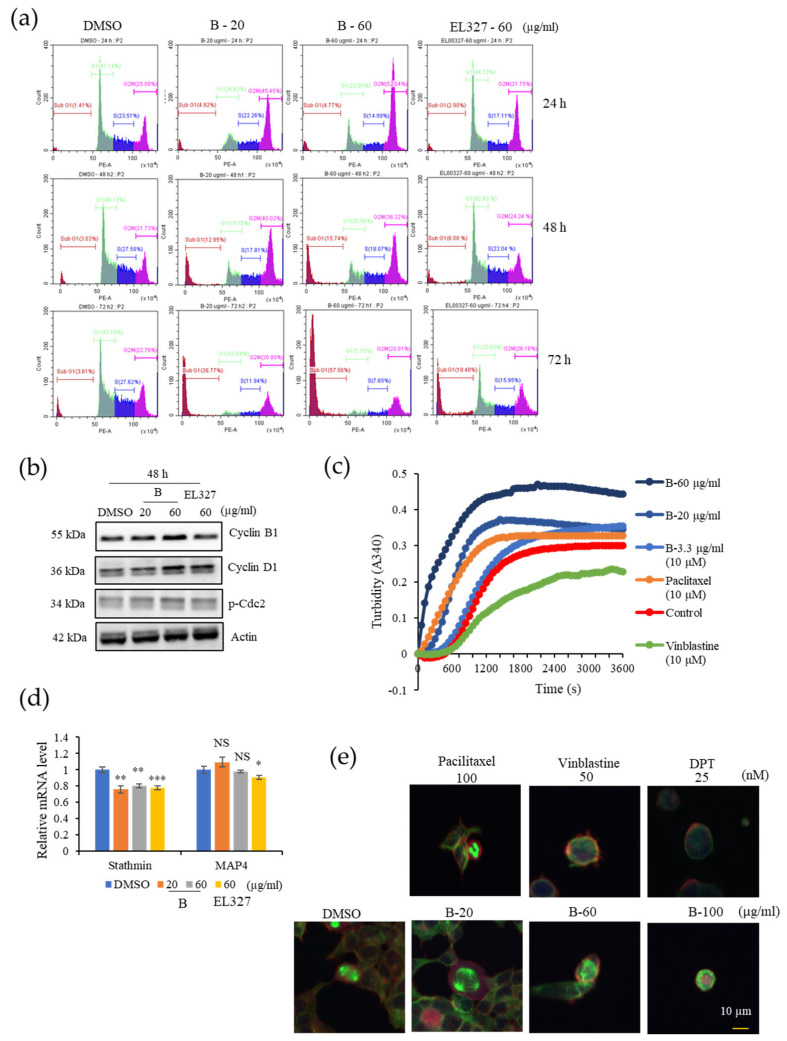
**B** induces G2/M phase arrest in Caco2 cells by inducing tubulin polymerization. (**a**) The cell-cycle distribution of Caco2 cells treated with **B** (20, 60 µg/mL) or EL000327 (60 µg/mL) for 24 h, 48 h, and 72 h as assessed by flow cytometry. (**b**) Western blot analysis of cell cycle regulating proteins, Cyclin B1, D1, and p-Cdc2 after treatment with **B** (20, 60 µg/mL) or EL000327 (60 µg/mL) for 24 h, 48 h, and 72 h. (**c**) Effect of **B** on tubulin polymerization in vitro, at concentrations of 20, 60, and 3.3 µg/mL. DMSO, paclitaxel, microtubule stabilizer (10 µM), and vinblastine microtubule destabilizer (10 µM) were used as the controls. (**d**) Relative mRNA levels of stathmin and MAP4, which are associated with microtubule destabilization and stabilization, respectively, after treatment with **B** (20, 60 µg/mL) or EL000327 (60 µg/mL) for 48 h. (**e**) Immunofluorescence microscopy of the microtubule organization in the Caco2 cells after treatment with **B** (20, 60, 100 µg/mL), paclitaxel (100 nM), vinblastine (50 nM) or DPT microtubule destabilizer (25 nM) for 24 h. Actin was stained with Alexa Fluor 568 phalloidin (red), microtubules were stained with α-tubulin antibodies (green), and DNA was stained with DAPI (blue). Results are representative of three independent experiments. Data represent the mean ± S.D. * *p* < 0.05, ** *p* < 0.01, *** *p* < 0.001, NS: no significant difference (*p* > 0.05) compared with the DMSO-treated control group.

To determine whether **B** inhibits CRC cell growth by regulating the cell cycle, the cell cycle distribution of Caco2, HCT116, DLD1, and HT29 cells was analyzed by flow cytometry after treatment with **B**. Caco2 cells were treated with cytotoxic concentrations of **B** (20, 60 µg/mL) or EL000327 (60 µg/mL) for 24 h, 48 h, and 72 h. **B** markedly increased the proportion of cells at the G2/M phase in a dose-dependent manner after 24 h of treatment. At 48 h and 72 h after treatment, the proportion of cells at the G2/M phase decreased in a time-dependent manner, accompanied by an increase in the sub G1 population, indicating cell death after G2/M phase arrest. Treatment with EL000327 caused some accumulation of cells in the G2/M phase, but to a lesser extent than the treatment with **B** (Figure 2a and Figure S1a). Analysis of the expression of known cell cycle regulatory proteins by Western blotting demonstrated that the expression of Cyclin B1, Cyclin D1, and p-Cdc2 was upregulated after treatment with **B** or EL000327 for 48 h (Figure 2b and Figure S5a). To compare the effect of **B** on cell cycles of other CRC cell lines, the HCT116, DLD1, and HT29 cells were treated with 20 µg/mL of **B** for 24 h and the distribution of the cell cycle was analyzed. Cells accumulated in the G2/M phase was increased in all cell lines compared to the control (Figure S1b,c).

The dynamics of microtubules play a vital role in the progression of the cell cycle through the G2/M phase because microtubules form mitotic spindles, which provide structural support for chromosome segregation during mitosis. Therefore, tubulin polymerization assays were performed to evaluate the effect of **B** on tubulin polymerization in vitro. Treatment with the well-known microtubule stabilizer paclitaxel (10 µM) enhanced tubulin polymerization, whereas the microtubule destabilizer, vinblastine (10 µM) impaired tubulin polymerization. In untreated conditions, microtubules self-assembled to form tubulin polymers in a time dependent manner. Treatment with **B** (3.3, 20, 60 µg/mL) had a similar effect to paclitaxel and enhanced tubulin polymerization in a dose- and time-dependent manner (Figure 2c). qRT-PCR analysis demonstrated that the tubulin destabilizing gene *Satathmin* was significantly downregulated upon treatment with **B** or EL000327 (Figure 2d). The changes in mitotic spindle organization in Caco2 cells seen after treatment with **B** were visualized by immunofluorescence staining to confirm the results described above. Immunofluorescence microscopy demonstrated that **B** had a similar effect on the microtubules to paclitaxel (100 nM) by inducing multipolar mitotic spindles as a result of enhanced tubulin polymerization. Treatment with high concentrations of **B** (20, 60, 100 µg/mL) increased the bundling and stabilization of microtubules in Caco2 cells in a dose dependent manner. In contrast, the known microtubule destabilizers, vinblastine (50 nM) and DPT (25 nM), resulted in disrupted microtubule organization (Figure 2e). These data suggest that **B** induced G2/M phase arrest by regulating the cell cycle related protein and by stabilizing the microtubules. Furthermore, **B** was identified as a microtubule stabilizing agent, which has a functional effect that is similar to that of paclitaxel.

### 3.3. **B** Induces Apoptotic Cell Death in CRC Cells

**Figure 3:**
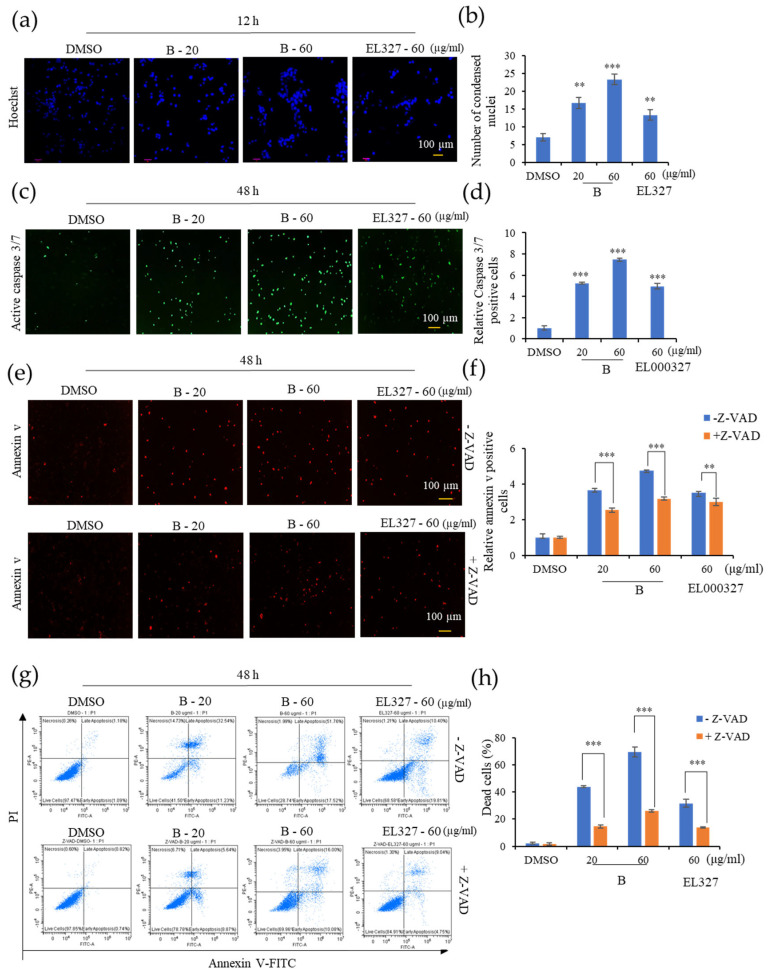
**B** induces caspase dependent apoptosis in Caco2 cells. (**a**) Nuclei condensation of Caco2 cells upon treatment with **B** (20, 60 µg/mL) or EL000327 (60 µg/mL) for 12 h, as determined by Hoechst staining. Arrowheads indicate nuclear condensation in cells. (**b**) Quantification of condensed nuclei in Caco2 cells treated with indicated concentrations of **B** or EL000327. (**c**) Caspase 3/7 (green) staining of Caco2 cells treated with **B** (20, 60 µg/mL) or EL000327 (60 µg/mL) for 48 h. (**d**) Quantification of apoptotic cells stained with Caspase 3/7 after treatment with the indicated concentrations of **B** or EL000327. (**e**) Annexin V staining of Caco2 cells treated with **B** (20, 60 µg/mL) or EL000327 (60 µg/mL) for 48 h in the presence or absence of the caspase inhibitor Z-VAD-FMK (10 µM). (**f**) Quantification of apoptotic cells stained with Annexin V after treatment with the indicated concentrations of **B** or EL000327 in the presence or absence of Z-VAD-FMK (10 µM). (**g**) Flow cytometric analysis of dead cells stained by Annexin v-FITC (apoptotic cells) and PI (necrotic cells) upon the treatment of **B** (20, 60 µg/mL) or EL000327 (60 µg/mL) for 48 h in the presence or absence of Z-VAD-FMK (10 µM). (**h**) Quantification of the percentage of apoptotic cells treated with indicated concentrations of **B** and EL000327 and analyzed by flow cytometry in the presence or absence of Z-VAD-FMK (10 µM). Results are representative of three independent experiments. Data represent the mean ± S.D. ** *p* < 0.01, *** *p* < 0.001; compared with the DMSO-treated control or Z-VAD-FMK treated group.

We next wished to determine whether the cytotoxicity exerted by **B** on CRC cells was due to the induction of apoptosis. Thus, Caco2, HCT116, DLD1, and HT29 cells were stained with Hoechst 33258 after treatment with cytotoxic concentrations of **B** or EL000327 for 12 h or 24 h to examine the morphological changes in the nuclei of cells. Cells treated with both **B** and EL000327 exhibited condensed chromatin, indicative of the initiation of apoptosis (Figure 3a and Figure S2a,b). The number of condensed nuclei significantly increased in all CRC cell lines (Figure 3b and Figure S2c). Furthermore, cell death induced by treatment with **B** was analyzed using the IncuCyte^TM^ apoptosis assay, which employs Caspase 3/7 (green) and Annexin V (red) dyes, in the presence or absence of the caspase inhibitor, Z-VAD-FMK. The level of Caspase-3/7 fluorescent green signal in Caco2 cells was markedly increased upon treatment with **B** or EL000327 for 48 h (Figure 3c,d). In this assay, the Annexin V dye emits a red fluorescent signal upon binding to the exposed phosphatidylserines (PS) of apoptotic cells. The number of red-labeled apoptotic cells increased after exposure to **B** in a dose-dependent manner. However, the suppression of caspases by Z-VAD-FMK significantly decreased the number of apoptotic cells detected by Annexin V staining (Figure 3e,f). To confirm the induction of caspase dependent apoptosis by **B** in Caco2 cells, flow cytometric analysis of apoptosis was also carried out. Cells were double stained with PI and Annexin V following treatment with **B** or EL000327 in the presence or absence of Z-VAD-FMK. Dose-dependently increasing numbers of apoptotic cells were observed after 48 h of treatment. Moreover, the inhibition of caspase significantly decreased apoptosis in the Caco2 cells (Figure 3g,h and Figure S3a). In addition, the flow cytometric analysis revealed that **B** significantly induced the apoptosis of other CRC cell lines HCT116, DLD1, and HT29 as well as at the concentration of 20 µg/mL (Figure S3b,c). Taken together, both **B** and EL000327 induced apoptotic cell death in CRC cells.

### 3.4. **B** Induces Caspase-Dependent Apoptosis and Autophagy in Caco2 Cells

**Figure 4:**
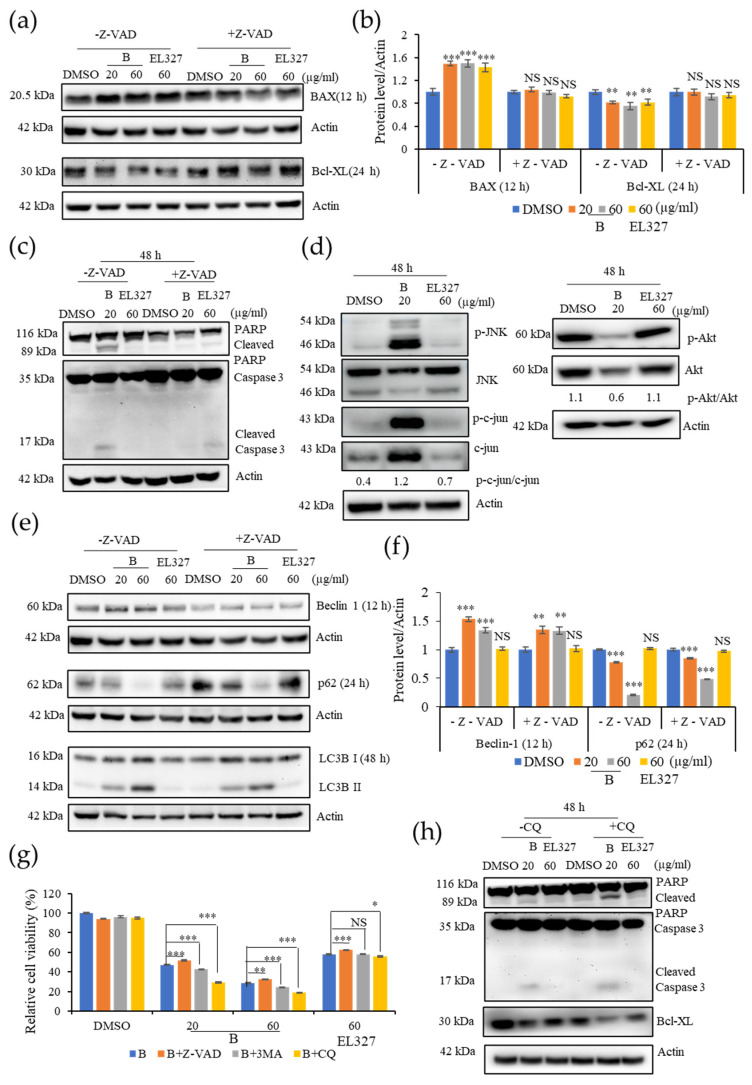
Inhibition of **B** induced autophagy increases apoptosis in Caco2 cells. (**a**) Western blot analysis of the pro-apoptotic protein BAX and the anti-apoptotic protein Bcl-xL treated by **B** (20 or 60 µg/mL) or EL000327 (60 µg/mL) for 12 or 24 h in the presence or absence of Z-VAD-FMK (10 µM). (**b**) Quantification of BAX and Bcl-XL protein expressions. (**c**) Western blot of apoptotic proteins; PARP, Caspase-3treated by **B** (20 or 60 µg/mL) or EL000327 (60 µg/mL) for 48 h in the presence or absence of Z-VAD-FMK. (**d**) Expressions of the apoptotic signaling pathway related proteins p-JNK, JNK, p-c-jun, c-jun, p-AKT, and AKT in Caco2 cells treated with **B** (20 µg/mL) or EL000327 (60 µg/mL) for 48 h, as analyzed by Western blotting. (**e**) Western blot of autophagy related proteins; Beclin 1 (12 h), p62 (24 h), and LC3BI/II (48 h) in Caco2 cells pre-incubated with or without Z-VAD-FMK, and treated with **B** (20, 60 µg/mL) or EL000327 (60 µg/mL). (**f**) Quantification of Beclin 1 and p62 protein expressions. (**g**) The relative percentage cell viability of Caco2 cells treated with **B** (20, 60 µg/mL) or EL000327 (60 µg/mL) for 48 h, with or without Z-VAD-FMK (10 µM) and autophagy inhibitors 3 MA (1 mM) and CQ (10 µM). (**h**) Expression levels of PARP, caspase-3, and Bcl-xL determined by Western blot analysis after treatment with **B** (20, µg/mL) or EL000327 (60 µg/mL) for 48 h in the presence or absence of CQ (10 µM). Data represent the mean ± S.D. * *p* < 0.05, ** *p* < 0.01, *** *p* < 0.001; NS: no significant difference (*p* > 0.05), compared with the Z-VAD-FMK, 3MA, and CQ treated groups or the DMSO-treated control.

Activation of the caspase dependent apoptosis by **B** in Caco2 cells was further confirmed by Western blot analysis in the presence or absence of Z-VAD-FMK. The level of the pro apoptotic protein BAX was significantly increased upon treatment with **B** or EL000327 at cytotoxic concentrations for 12 h. However, a significant change in the levels of BAX were not observed in cells that had been pretreated with Z-VAD-FMK. In contrast, the expression level of the anti-apoptotic protein Bcl-xL was decreased in the Caco2 cells after treatment with **B** or EL000327 for 24 h, but no change in the presence of Z-VAD-FMK (Figure 4a,b). Clear cleavage of the main apoptotic markers PARP and caspase-3 was detected after treatment with **B** (20, 60 µg/mL) or EL000327 (60 µg/mL) for 48 h in the absence of Z-VAD-FMK (Figure 4c). The effect of **B** and EL000327 on the activation of the JNK/c-jun and Akt signaling pathways was assessed, as these pathways eventually lead to the induction of apoptosis as well as autophagy in cells. Phosphorylation of JNK and c-jun was markedly induced upon treatment with **B** at IC_50_ concentrations for 48 h and decreased the phosphorylation of Akt. However, no significant changes in protein levels were detected in cells treated with EL000327 (Figure 4d). Regulation of the autophagy related proteins Beclin 1, p62, and LC3BI/II was also examined in the presence or absence of Z-VAD-FMK. Upregulation of Beclin 1 and LC3BI/II and downregulation of the p62 protein levels were observed in Caco2 cells upon treatment with cytotoxic concentrations of **B** or EL000327, indicating the inhibition of autophagosome degradation in these cells. However, consistent regulation patterns were not observed for these autophagy markers in the presence of Z-VAD-FMK (Figure 4e,f).

In order to confirm the major cell death pathway induced by **B** and crude extract EL000327, cell viability was assessed after pretreatment with apoptotic and autophagic inhibitors. Treatment with Z-VAD-FMK significantly increased the cell viability in Caco2 cells in response to treatment with **B** or EL000327. In contrast, the application of 3MA (3-methylladenine), a blocker of autophagosome formation, and CQ (chloroquine), an inhibitor of lysosomal acidification and autophagosome degradation, resulted in a dose dependent decrease in cell viability, indicating that the inhibition of autophagy enhanced the cytotoxicity of **B** toward Caco2 cells (Figure 4g). Of the autophagy blockers, CQ had a more potent effect on the induction of cell death than 3MA. Application of CQ markedly increased the levels of cleaved PARP and caspase-3 and decreased the level of Bcl-xL in the cells treated with **B** for 48 h (Figure 4h and Figure S5b). These data suggest that **B** induced caspase dependent apoptosis mainly via the activation of the JNK/c-jun pathway in Caco2 cells. Furthermore, the observed induction of autophagy by **B** may activate the protective mechanism in cells.

### 3.5. **B** Activates JNK/c-Jun Signaling Pathway via Triggering ROS Generation

**Figure 5:**
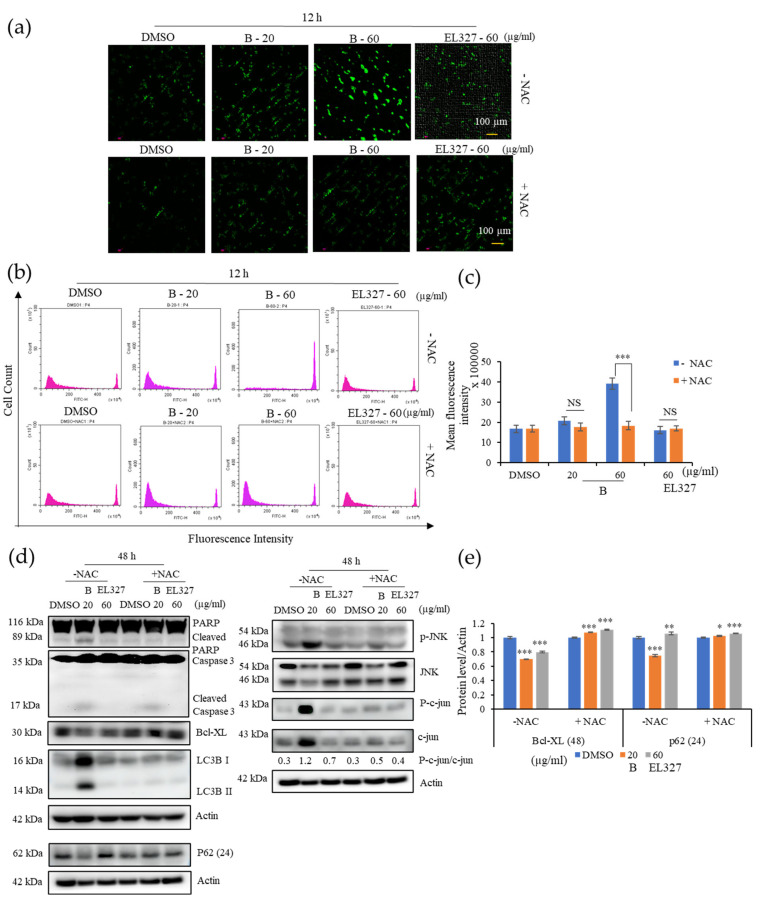
**B** induces ROS generation and activates JNK signaling in Caco2 cells. (**a**) Intracellular ROS generation was detected by fluorescence microscopy using DCFH-DA (10 µM) in Caco2 cells treated with **B** (20, 60 µg/mL) or EL000327 (60 µg/mL) for 12 h with or without the ROS inhibitor NAC (5 mM). (**b**) Flow cytometric analysis of the fluorescence intensity of Caco2 cells preincubated with DCFH-DA (10 µM) and treated with **B** (20, 60 µg/mL) or EL000327 (60 µg/mL) for 12 h with or without NAC (5 mM). (**c**) Quantification of the mean fluorescence intensity of Caco2 cells preincubated with DCFH-DA (10 µM) and treated with the indicated concentrations of **B** for 12 h in the presence or absence of NAC. (**d**) Western blot analysis of PARP, caspase-3, Bcl-xL, p62, LC3BI/II, p-JNK, JNK, p-c-jun, and c-jun protein expression after treatment with **B** (20, µg/mL) or EL000327 (60 µg/mL) for 24 or 48 h, with or without NAC (5 mM). (**e**) Quantification of Bcl-XL and p62 protein expressions in the presence or absence of NAC. Data represent the mean ± S.D. * *p* < 0.05, ** *p* < 0.01, *** *p* < 0.001; NS: no significant difference (*p* > 0.05) compared with the NAC-treated group or the DMSO-treated control.

Intracellular ROS play a vital role in activating cellular apoptotic and autophagic functions. To assess the effect of **B** on ROS generation, cells were treated with **B** or EL000327 at toxic concentrations for 12 h in the presence or absence of the antioxidant N-acetyl cysteine (NAC), and ROS were detected by staining cells with DCFH-DA, followed by fluorescence microscopy or flow cytometry. While exposure of Caco2 cells to **B** dramatically increased the green fluorescent signals indicative of ROS generation, pretreatment with NAC significantly reduced the fluorescent signals, suggesting a block in ROS generation (Figure 5a). The number of fluorescently labeled cells decreased upon treatment with NAC, as detected by flow cytometry. No significant change in fluorescence emission was detected in the EL000327 treated cells after 12 h compared to the control cells (Figure 5b,c). Thus, pretreatment with NAC decreased the expression of apoptotic and autophagy markers and rescued cells from **B**-induced apoptosis and autophagy. Furthermore, NAC reversed the phosphorylation of JNK and c-jun in cells treated with IC_50_ concentrations of **B** (Figure 5d,e). Collectively, these results indicate that **B** activates the ROS/JNK signaling pathway in Caco2 cells.

### 3.6. **B** Induces Apoptosis and Autophagy in Caco2 Cells by Activating ROS/JNK Signaling Pathways

**Figure 6:**
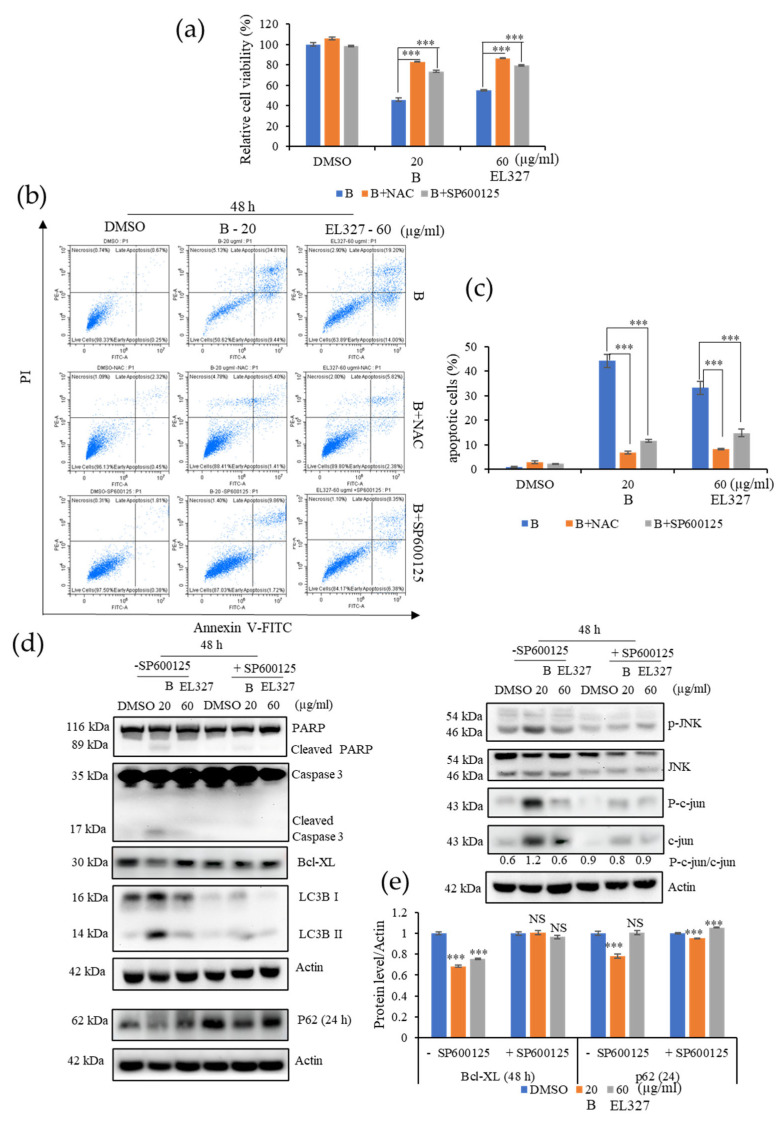
**B** activates ROS/JNK mediated apoptosis and autophagy in Caco2 cells. (**a**) The relative percentage cell viability of Caco2 cells detected after treatment with **B** (20 µg/mL) or EL000327 (60 µg/mL) for 48 h, with or without NAC (5 mM) or the JNK inhibitor SP600125 (10 µM). (**b**) Flow cytometric analysis of dead cells stained by Annexin V-FITC and PI after treatment with **B** (20 µg/mL) or EL000327 (60 µg/mL) for 48 h, with or without NAC (5 mM) or SP600125 (10 µM). (**c**) Quantification of the percentage of apoptotic cells after treatment with the indicated concentrations of **B** or EL000327 for 48 h with or without NAC and SP600125 and analyzed by flow cytometry. (**d**) Western blot analysis of apoptosis, autophagy, and the JNK signaling pathway related protein expression in Caco2 cells treated with **B** (20 µg/mL) or EL000327 (60 µg/mL) for 24 or 48 h, with or without SP600125 (10 µM). (**e**) Quantification of Bcl-XL and p62 protein expressions in the presence or absence of SP600125. Data represent the mean ± S.D. *** *p* < 0.001 compared with the NAC- and SP600125-treated groups or the DMSO-treated control. NS: no significant difference.

The involvement of ROS and JNK activation for the induction of apoptosis and autophagy by **B** and EL000327 was further investigated. Pretreatment of Caco2 with NAC or the JNK inhibitor SP600125 significantly reduced the sensitivity to **B** and EL000327 compared to the cells treated with **B** or EL000327 in the absence of inhibitors (Figure 6a). Flow cytometric analysis detected very low numbers of apoptotic cells in the presence of NAC or SP600125, confirming that ROS and JNK inhibitors rescued **B**-induced cell death in the Caco2 cells (Figure 6b,c and Figure S4a). Moreover, the application of SP600125 significantly decreased the expression of apoptotic and autophagy markers as well as JNK pathway related proteins, as analyzed by Western blotting after treatment with **B** for 24 or 48 h (Figure 6d,e). Taken together, these results reveal that triggering the generation of ROS by **B** activates JNK/c-jun signaling and leads to the induction of apoptosis and autophagy in Caco2 cells.

### 3.7. **B** Exhibits Synergy with 5-FU and Compound D on CRC Cells

**Figure 7:**
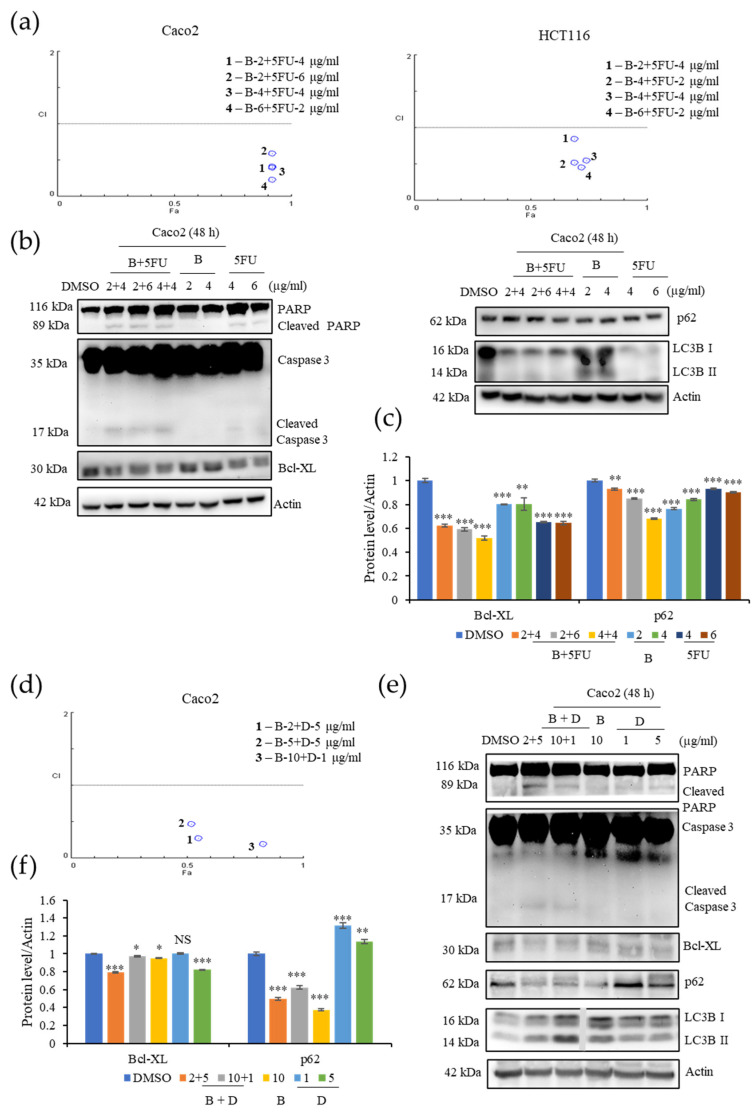
**B** shows synergy with 5-FU and with D, a compound isolated from the crude extract of EL000327 on the CRC cells. (**a**) Fa-CI plot of combination treatment with 5-FU (2, 4 or 6 µg/mL) and **B** (2, 4 or 6 µg/mL) on the Caco2 cells and the Fa-CI plot of combination treatment with 5-FU (2 or 4 µg/mL) and **B** (2, 4 or 6 µg/mL) on the HCT116 cells. (**b**) Expression of apoptosis and autophagy related protein in Caco2 cells after combination treatment with 5-FU (4 or 6 µg/mL) and **B** (2 or 4 µg/mL) or **B** (2 or 4 µg/mL) or 5FU (4 or 6 µg/mL) individually for 48 h, as detected by Western blotting. (**c**) Quantification of Bcl-XL and p62 protein expression after the treatment with **B**+5FU or **B** or 5FU at the indicated concentrations. (**d**) Fa-CI plot of combination treatment with D (5 or 1 µg/mL) and **B** (2, 5 or 10 µg/mL) on the Caco2 cells. (**e**) Western blot analysis of apoptosis and autophagy related protein expression in the Caco2 cells after combination treatment with D (5 or 1 µg/mL) and **B** (2 or10 µg/mL) or **B** (10 µg/mL) or D (1 or 5 µg/mL) individually for 48 h. (**e**) Quantification of Bcl-XL and p62 protein expression after the treatment with **B** + D or **B** or D at the indicated concentrations. Data represent the mean ± S.D. * *p* < 0.05, ** *p* < 0.01, *** *p* < 0.001; NS: no significant difference (*p* > 0.05) compared with the NAC-treated group or the DMSO-treated control.

To investigate the potential of **B** as an anticancer therapeutic drug in the clinic, we analyzed its effect when used in combination with 5-FU, a well-known chemotherapeutic that is used to treat several types of cancers including colorectal cancers, and D (IC_50_ = 10 μg/mL), another cytotoxic compound isolated from EL000327 (Figure 1c). The chemical structure of D is yet to be determined. Caco2 were treated with **B**, 5-FU, and D singly or 5-FU combined with **B** or D, and the HCT116 cells were treated with **B**, and 5-FU singly or 5-FU combined with **B** at various concentrations for 48 h; cell survival was analyzed using a MTT assay, The Chou–Talalay method was used to calculate the combination index (CI) of synergy using compuSyn software. Anti-cancer agents with synergism have a CI value of <1, and those with the smallest CI value are considered to be more suitable for cancer therapy. The combination of 2 μg/mL of **B** with 4 μg/mL (**1**; CI = 0.4) or 6 μg/mL of 5-FU (**2**; CI = 0.6), the combination of 4 μg/mL of **B** with 4 μg/mL of 5-FU (**3**; CI = 0.41), and the combination of 6 μg/mL of **B** with 2 μg/mL of 5-FU (**3**; CI = 0.23) all produced CI values of less than 1 on Caco2 cells. Furthermore, **B** exhibited good synergism with 5FU on HCT116 cells as well as when combined with 2 μg/mL of **B** with 4 μg/mL of 5-FU (**1**; CI = 0.96), 4 μg/mL of **B** with 2 μg/mL (**2**; CI = 0.52), or 4 μg/mL of 5-FU (**3**; CI = 0.54) and 6 μg/mL of **B** with 2 μg/mL of 5-FU (**4**; CI = 0.45) (Figure 7a). Western blot analysis demonstrated that the combination of **B** and 5-FU at the concentrations that provided a CI value of <1 considerably increased the cleaved PARP and Caspase-3expressions and decreased the level of anti-apoptotic protein Bcl-XL compared to treatment with **B** and 5-FU individually. Decreased level of p62 was observed after the combination treatments (Figure 7b,c). Furthermore, 2 μg/mL of **B** combined with 5 μg/mL (**1**; CI = 0.27), 5 μg/mL of **B** with 5 μg/mL of D (**2**; CI = 0.47), and 10 μg/mL of **B** with 1 μg/mL of D (**3**; CI = 0.19) (Figure 7d). Expression of cleaved PARP, caspase-3, and LC3BI/II was elevated when the cells were subjected to combined **B** and D treatments, as demonstrated by Western blotting. Furthermore, the levels of anti-apoptotic protein Bcl-XL and autophagy related protein p62 were downregulated by the combination treatment with **B** and D (Figure 7e,f). These results suggest that **B** acts synergistically with both 5-FU and compound D to significantly increase its cytotoxic effects on CRC cells at low concentrations. Therefore, **B** shows a high potential to act synergistically with other anticancer therapeutics to enhance their effect by increasing the cytotoxicity.

## 4. Discussion

Natural products have been highly recognized as rich reservoirs of bioactive compounds with the potential to lead to the discovery of novel anticancer therapeutics. As a result of the tremendous efforts by scientists, many naturally derived anticancer agents have been discovered and successfully developed into drugs within the last 30 years. Such compounds account for approximately 25% of newly approved anticancer drugs [25]. Lichen substances and the secondary metabolites of endolichenic fungi have also been proven to have a wide range of anticancer activities against various types of cancers [5,26,27,28,29,30,31]. Therefore, it is essential to carry out a thorough investigation of bioactive compounds derived from the rich bioresources of lichens. Libertellenone T **(B)** is a novel cytotoxic compound, isolated as a pink oil from a secondary metabolite extract of the endolichenic fungi EL000327. Its molecular formula was determined to be C_20_H_26_O_4_, based on HR-FAB-MS, coupled with the analysis of the NMR data. As the crude extract of EL000327 exerted comparatively high cytotoxicity on CRC, which is considered to be the second most lethal cancer type in the world [32], the cytotoxicity of **B** on CRC was assessed. Interestingly, the effect of **B** on the CRC cells was much stronger than EL000327 and the Caco2 cells were highly sensitive to the treatment of **B** compared to the HCT116, DLD1, and HT29 cells. Furthermore, the effect of **B** on the non-cancerous cell line Madin–Darby canine kidney epithelial cells (MDCK) was very low and the human non-cancerous cell line HaCaT was similar to the Caco2 cells. In the current study, the mechanisms underlying **B**-induced cell death were comprehensively investigated. We found that **B** induced mitotic arrest in CRC cells by stabilizing microtubules and preventing their depolymerization. Moreover, **B** activated apoptosis and autophagy via the ROS/JNK signaling pathway. **B**-induced autophagy had a protective effect on Caco2 cells. Most importantly, **B** showed synergy with the well-known chemotherapeutic 5-FU and another novel compound D isolated from the same crude extract of EL000327. Here, we mainly used Caco2 cells to study the mechanism of action of **B**, but our results revealed that **B** has an effect on other CRC cells such as HCT116, DLD1, and HT29. The induction of apoptosis in these cells upon the treatment of **B** was confirmed by the results of Hoechst staining and flow cytometry.

Check points prevent cells with damaged DNA from entering the next phase of the cell cycle. This phenomenon is highly regulated by a series of proteins, and the G2/M transition is mainly regulated by the Cyclin B/Cdc2 (Cdk1) complex. Phosphorylation of Cdc2 negatively regulates cell cycle progression from the G2 phase to M phase [11]. Furthermore, the Cyclin B/Cdk1 complex is highly activated in the metaphase as it supports the assembly of the mitotic apparatus and chromosome alignment. Once chromosomes are properly attached to spindles, the APC/C is activated and promotes progression to anaphase.

Degradation of cyclin B by the activation of the APC/C complex leads to Cdk1 inactivation [33]. Flow cytometric analysis of cell cycle progression in our study indicated a clear accumulation of Caco2 cells in the G2/M phase than other CRC cell lines HCT116, DLD1, and HT29 after treatment with **B** for 24 h. However, increasing G2/M cell populations compared to the control indicated that HCT116, DLD1, and HT29 cells require longer treatment to induce G2/M phase arrest. Furthermore, treatment with **B** resulted in the upregulation of Cyclin B1 and p-Cdc-2 expression. Therefore, we continued our studies to investigate whether **B** affected the microtubule dynamics in cells. According to the results of our in vitro tubulin polymerization assay, **B** stabilized microtubules in a manner similar to the clinically approved microtubule stabilizer, paclitaxel. These results were further confirmed by the immunofluorescence staining of cells treated with **B** and paclitaxel. Microtubule targeting agents in anticancer therapy can be classified into two groups based on their mode of action. Microtubule destabilizers prevent microtubule formation by inhibiting tubulin dimerization. In contrast, microtubule stabilizers promote tubulin dimerization and stabilize microtubules [34]. Both the stabilization and destabilization of microtubules lead to mitotic catastrophe followed by cell death due to failure to form the spindle required for chromosome segregation in the M phase of the cell cycle. Similarly, our compound, **B**, induced cell death after prolonged mitotic arrest, as shown by the appearance of a sub G1 population in cell cycle analysis.

Apoptosis is the process of programmed cell death and the most popular target of many anticancer therapies. Dysregulation of apoptosis signals in cancers promotes abnormal cell growth and tumorigenesis [9]. Restoring the lost apoptotic function in cancer cells is the main objective of much of the research into cancer treatments. The initiation of apoptosis in cells can be identified by morphological changes such as nuclear fragmentation, chromatin condensation, cell shrinkage, and membrane blebbing [35,36]. Hoechst, Caspase 3/7, and Annexin V staining demonstrated the initiation of apoptosis in CRC cells upon treatment with **B**. Apoptosis can be classed as either caspase-dependent or caspase-independent. Many anticancer agents mainly activate the caspase-dependent mitochondrial pathway. In addition, the death receptor mediated, and endoplasmic reticulum pathways also activate caspases at the final phase of apoptosis. Disruption of the mitochondrial membrane potential followed by translocation of AIF and endonuclease G to the nucleus induces caspase-independent apoptosis [37]. Data from the IncuCyte apoptosis assay and flow cytometric analysis of apoptosis demonstrated that treatment with the caspase inhibitor Z-VAD-FMK significantly reduced the extent to which **B** induced apoptosis in cells, indicating that apoptosis induced by **B** is caspase-dependent. Furthermore, Western blot analysis showed the activation of the proapoptotic protein BAX, the anti-apoptotic protein Bcl-xL, and eventually, Caspase-3and PARP cleavage in response to **B** treatment. Caspase-3 is the executioner caspase in all caspase dependent apoptosis pathways. Caspase-3 can be cleaved and activated by the upstream caspase, caspase-8, in the extrinsic pathway as well as by caspase-9, an initiating caspase, in the intrinsic pathway [38]. Cleavage of PARP by caspases is considered as a hallmark of apoptosis and an indicator that caspase dependent apoptosis has been accomplished [39]. Here, as predicted, Z-VAD-FMK treatment significantly impaired the expression of apoptotic markers in cells treated with **B**. Interestingly, the expression of the autophagic proteins Beclin 1 and LC3BI/II slightly decreased while p62 expression slightly increased upon treatment with **B** in the presence of Z-VAD-FMK. However, this result is not sufficient to assess the action of **B** toward autophagy in the presence of Z-VAD-FMK.

Autophagy plays a dual role in cancer treatment by either supporting or preventing cancer cell survival. In the current study, **B** induced autophagy in the Caco2 cells. Moreover, treatment with the autophagy inhibitors 3MA and CQ significantly increased **B**-induced cell death, suggesting that the activation of autophagy promotes CRC cell survival. Western blot analysis further confirmed that the inhibition of autophagy enhanced the activation of apoptosis, as demonstrated by elevated cleaved caspase-3 and PARP levels in the presence of CQ. Beclin 1, P62, and LC3BI/II are key regulators of the autophagic process. Beclin 1 regulates autophagosome formation at the beginning of autophagy. During autophagosome formation, cytosolic LC3B-I is converted to the membrane-bound LC3B-II form. Binding of LC3B to the adapter protein p62/SQSTM1 facilitates autophagic degradation [40]. In the present study, we observed increased the expressions of Beclin 1 and, LC3B-II and decreased p62 upon treatment with **B**. While elevated expression of Beclin 1 and, LC3B is indicative of the activation of autophagy in CRC cells, generally, activation of autophagy results in reduced expression of p62. However, under some circumstances, p62 expression can be elevated by upregulation of p62 transcription during starvation of cells, regardless of the effect of autophagy. Under prolonged starvation, p62 expression is restored to basal levels by transcriptional regulation, even, when its expression has been decreased by autophagic activities at earlier time points [41,42]. Furthermore, p62 transcription is modulated by oxidative stress (Nrf2), the Ras/MAPK pathway, and the JNK/c-Jun pathway as well as some chemicals, including autophagy inducers [43]. Following treatment with **B**, cell viability was lower in the presence of CQ than 3MA. Furthermore, the level of LC3B-II was significantly increased upon treatment with CQ in western blot analysis. CQ inhibits autophagy by inhibiting fusion of the autophagosome and lysosome, and by degradation of the autophagolysosome [44]. These results suggest that **B** may induce autophagy by promoting late phase autophagy, fusion, and degradation. Moreover, the expression of autophagy related proteins was decreased when apoptosis was suppressed by Z-VAD-FMK. In contrast, expression of apoptotic markers was increased when autophagy was blocked by CQ. This leads to the hypothesis that **B** predominantly induces apoptosis in CRC cells, and that autophagy is activated as a counter mechanism to protect the cells from apoptosis. However, this potential interconnection between activation of apoptosis and autophagy by **B** needs further, detailed investigation.

Our study revealed that ROS generation is highly induced upon the treatment with **B**. Excess levels of ROS can be deleterious to cells due to the induction of oxidative stress within cells. Blocking ROS generation using the antioxidant NAC decreased expression of both apoptotic and autophagy markers in response to treatment with **B**, as demonstrated by western blot analysis. Furthermore, NAC significantly decreased the phosphorylation of JNK and expression of c-JUN. Given that ROS generation began after 12 h of treatment with **B**, while apoptotic markers were detected after 48 h, it seems likely that **B** induced apoptosis is initiated by ROS. ROS activate many signaling pathways (PI3K/Akt, MAPK, Nrf2) and transcription factors (NF-κB, p53) that eventually induce apoptosis, autophagy, or necrosis. Oxidants like OH^•^, ONOO^−^, and H_2_O induce apoptosis and/or necrosis, while O_2_^•−^ and H_2_O_2_ induce autophagy and mostly trigger cell survival.

**Figure 8:**
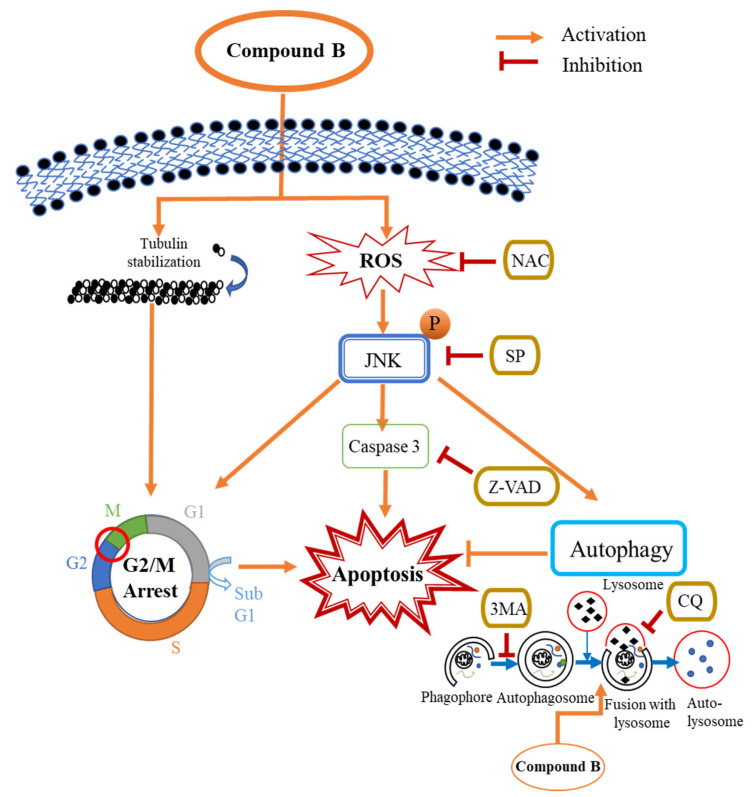
Schematic representation of the proposed mechanism for **B**-induced apoptosis and autophagy in the Caco2 cells. **B** induces apoptosis in Caco2 cells through the induction of G2/M phase arrest caused by tubulin stabilization. Simultaneously, **B** induces apoptosis and autophagy in Caco2 cells via the ROS/JNK signaling pathway. Inhibition of ROS, JNK, and caspases by NAC, SP600125, and Z-VAD-FMK, respectively, decreases **B** induced caspase-dependent apoptosis in Caco2 cells. 3MA and CQ inhibit autophagy in the Caco2 cells. The inhibition of autophagy by preventing the fusion of lysosomes with autophagosomes by CQ significantly increases the induction of caspase-dependent apoptosis by **B** in the Caco2 cells.

Cell viability upon treatment with **B** was markedly increased in the presence of the NAC and JNK inhibitor, SP600125, while apoptosis was significantly decreased, according to flow cytometric and western blot analysis. Furthermore, levels of LC3B were also reduced in the presence of SP600125, indicating that autophagy was inhibited. Taken together, this suggests that JNK plays a significant role in the apoptosis and autophagy activated by treatment with **B**. Induction of c-jun/JNK signaling by ROS blocks the antiapoptotic protein, Bcl-2, and activates the proapoptotic proteins in the Bcl-2 family, which are critical for the release of cytochrome c to the cytosol. Activation of caspase-9 and the effector caspase-3 by cytochrome c eventually leads to cell death via the mitochondrial apoptotic pathway. ROS can also activate the extrinsic apoptotic pathway by directly causing damage to DNA [45]. Taken together, the above results suggest that **B** induces apoptosis and autophagy in CRC via activation of ROS/JNK signaling (Figure 8).

EL000327 is the crude extract from which **B** was isolated. This crude extract contained six compounds, including **B** and another highly cytotoxic compound, D. In our study, we compared the effect of our isolated compound on CRC cells with that of the crude extract, EL000327. Initiation of apoptosis upon treatment with EL000327 was detected by Hoechst staining, the incucyte apoptosis assay, and flow cytometric analysis of apoptosis. However, apoptotic markers were not clearly detected by western blot analysis at the same time points as **B**. Induction of cell cycle arrest, activation of autophagy, or the ROS/JNK pathway related proteins in Caco2 cells were also not observed upon treatment with EL000327. These results suggest that interactions between different compounds in the crude extract may alter the activity of EL000327. Alternatively, the effects of EL000327 and **B** may have been detected at different time points because EL000327 was either faster or slower to act than **B**.

*5-Fluorouracil* (*5-FU*) is a first-line treatment for many cancers, including CRC. 5-FU leads cells to death by preventing DNA replication and RNA synthesis through inhibition of cellular thymidylate synthase (TS) [46,47]. Combination treatments in which 5-FU is combined with different anticancer agents, enhance the anticancer effect and response rate of these treatments. Similarly, combination of **B** with 5-FU enhanced the cytotoxicity of **B** toward Caco2 and HCT116 cells at significantly lower treatment concentrations [48,49]. Combination of **B** and 5-FU induced apoptosis in Caco2 cells by enhancing the expression of cleaved caspase-3and PARP. Further investigations are required to understand the mechanism by which cytotoxicity is increased in the synergy between **B** and 5-FU. Furthermore, **B** exhibited excellent synergic effects with compound D. A combination of **B** and D induced apoptosis and autophagy in the Caco2 cells at comparatively low concentrations. These results provide evidence that **B** may act synergistically with a range of different anticancer agents to enhance their cytotoxic effect.

**B** is a novel naturally-derived compound, and this is the first study of the activity of **B** on CRC cells. We thoroughly investigated the mechanisms by which **B** induces apoptotic cell death and have laid the groundwork for a detailed analysis of the clinical usefulness of **B.** Moreover, we have demonstrated that the combination of **B** with known and novel anticancer agents may provide effective new treatment options for patients with CRC, which warrants further investigation.

## 5. Conclusions

The results of this study demonstrate that **B** induced caspase dependent apoptosis and autophagy in Caco2 cells via activating the ROS/JNK signaling pathway in vitro. In addition, **B** stabilized the microtubule dynamics and disrupted cell cycle progression by inducing G2/M phase arrest. Moreover, **B** exhibited synergism with known chemotherapeutic 5FU and novel compound D on the CRC cells. Together, **B** shows great potential as a novel drug candidate that can be used for further research to develop into an anticancer therapeutic against CRC.

## Data Availability

All of the data generated or analyzed during this study are included in the published article and its Supplementary files.

## Supplementary Materials

The following supporting information can be downloaded at: https://www.mdpi.com/article/10.3390/cancers15020489/s1, Table S1: Nucleotide sequence of EL000327. Figure S1: B induces G2/M phase arrest in the CRC cells; Figure S2: **B** induces nuclei condensation in the CRC cells; Figure S3: **B** induces apoptosis in CRC cells; Figure S4: **B** decreased apoptosis in the Caco2 cells in the presence of ROS and JNK inhibitors; Figure S5: **B** modulated cell cycle related protein expressions and anti-apoptotic protein Bcl-XL; Figure S6: ^1^H NMR spectrum of Libertellenone T (**B)** in MeOD; Figure S7: ^13^C NMR spectrum of Libertellenone T (**B)** in MeOD; Figure S8: Percentage purity of Libertellenone T (**B**); Figure S9: The raw data of Western blotting.
