# Supplementary material for: Libertellenone T, a Novel Compound Isolated from Endolichenic Fungus, Induces G2/M Phase Arrest, Apoptosis, and Autophagy by Activating the ROS/JNK Pathway in Colorectal Cancer Cells
Source: Cancers (Basel). 2023 Jan 12;15(2):489. doi: 10.3390/cancers15020489 (PMC9857212; doi:10.3390/cancers15020489)
Supplement: Supplementary file 1 [file cancers-15-00489-s001.zip › cancers-2025688-supplementary.pdf]

Libertellenone T, a Novel Compound Isolated from Endolichenic Fungus, Induces G2/M Phase Arrest, Apoptosis, and Autophagy by Activating the ROS/JNK Pathway in Colorectal Cancer Cells

Chathurika D. B. Gamage [1], Jeong-Hyeon Kim [2], Yi Yang [1], İsa Taş [1], So-Yeon Park [1], Rui Zhou [1], Sultan Pulat [1], Mücahit Varlı [1], Jae-Seoun Hur [3], Sang-Jip Nam [2,*] and Hangun Kim [1,*]

Supplementary Table S1

Supplementary Figure S1–S9

Table S1. Nucleotide sequence of EL000327

CACAGCTAGAGGGCAAGGACCATCAGAAGCGAATAAATATCACGCCGAGGTCTCTTGCAG
CTTCTGCTGAAGGATTTAAGAGGGGATCGACCAAGGTTGATCCTCCTCCAGGTCCAGGTGC
AATGCACCTGAGGATGTGTTTGATGACGCTCGAACAGGCATACCTCCCGGAATACCAGGA
GGTGCAATGTGCGTTCAAAGATTCGATGATTCACTGAATTCTGCAATTCACACTACTTATCG
CATTTCGCTGCGTTCTTCATCGATACGAGAACCAAGAGATCCGTTGTTGAAAGTTTTAACTT
GTTTGCGGTGTTGTTAACACTCGGACTGCCACGATGGAAATAACGAGGGTTTAAACGCTAT
TACCGTGCGCCCTTGGGACGTAACCCCAAGGCTTTTCAGGGAGCAACCTCCGTTACCCGGG
TAGGGTCAGAGGAAGCTGCAGGCAACACGGAAGCAACAGGAGAATGTTCACAGGAGGGA
GGAAATCAAATGATCACTTGAAGAGGATTCTTGATCCGTCCATAAGATGAAGGTATAGATG
GAGGTCTTCGCCTAGCTGCAAGCTTGCGACGTCAATGCAACCCACCCACAGTCTAGGGTTT
GACCCCTCCGGACTCAGCGGGTACCCACCCCTAGTTCCAGCAGATGCAGAGCAGAGCTAC
TGGGACACAGAGGATTATGGGGTAGGGTGACGCCACCCGATCGGTTTACGCAAAGTGCCC
ACGTACAAGCATCTATGCCACGACCCCGGTTAATCCTAGGCTTCCACGTTTGCCAGACTCG
ATTCCCCAGTGGGAATCGGTGGGGACAGACAGCCAGTGGACTCAGCAGCGTTAACTGCCC
ATCCCTTGCAGCCCTACTATGGACAGGTGACACCACCCACAGCCATAGTAGCCCAAGGCA
ATCCCCTTACCACACTATCTGTTCCACGGCATCATGTTCAAGTATAGTGATGGTCAAGAGAT
CTCAGATTGCCTCGATCGACTGATCTCCATGCCGATCCCATGGGCAGGACCCTCTTCAAGC
ATTGCACTTTATTTAATGATCCTTCCGCAGGTTCACCTAC

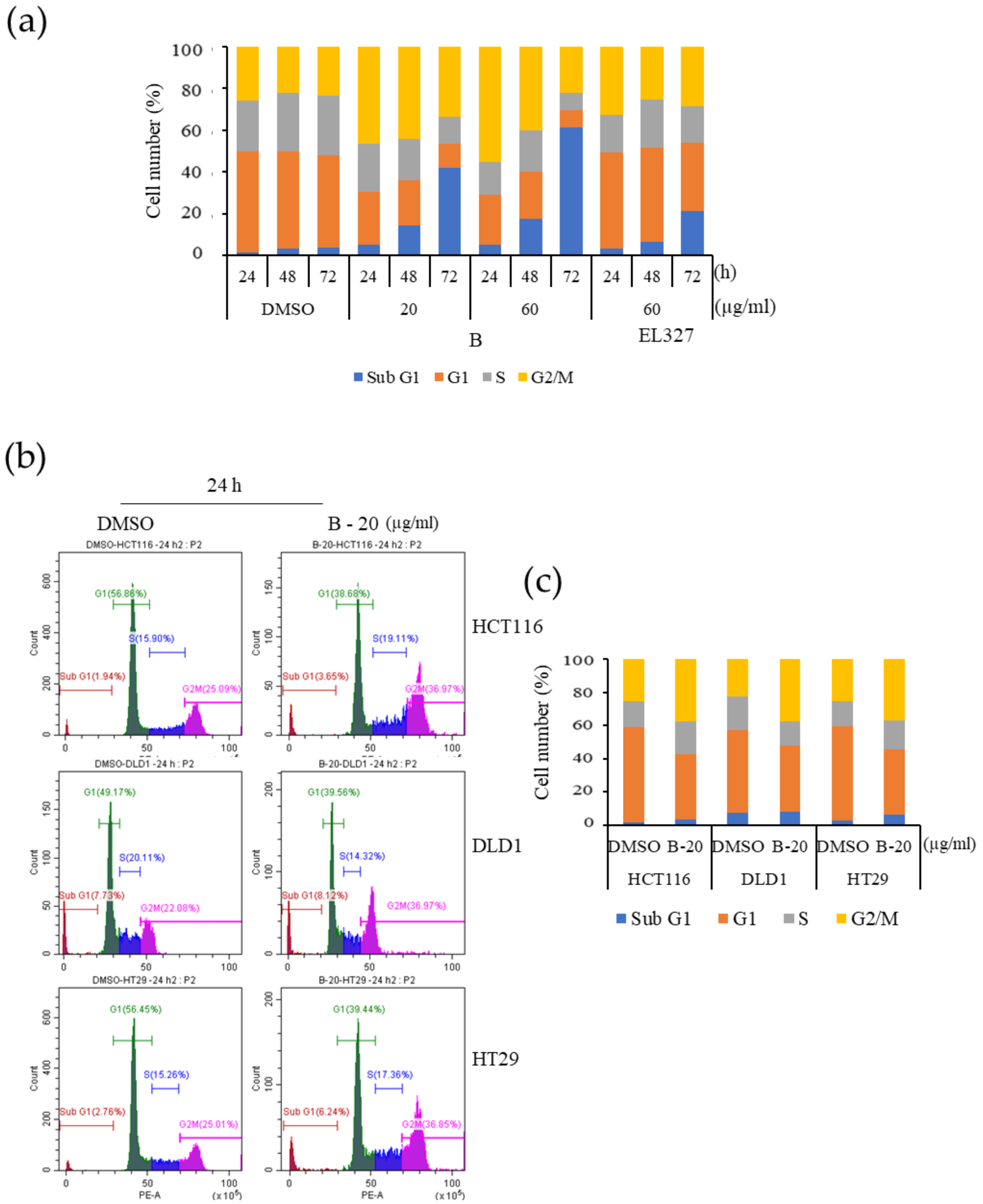

Figure S1. **B** induces G2/M phase arrest in CRC cells. (a) Quantitative analysis of percent cell distribution in each phase of the cell cycle upon the treatment of 20, 60 μg/ml of **B** and 60 μg/ml of EL000327 for 24, 48 and 72 h. (b) The flow cytometric analysis of the cell-cycle distribution of HCT116, DLD1 and HT29 cells treated with 20 μg/ml of **B** for 24 h. (c) Quantitative analysis of percent cell distribution in each phase of the cell cycle upon the treatment of 20 μg/ml of **B** for 24 h. n=3.

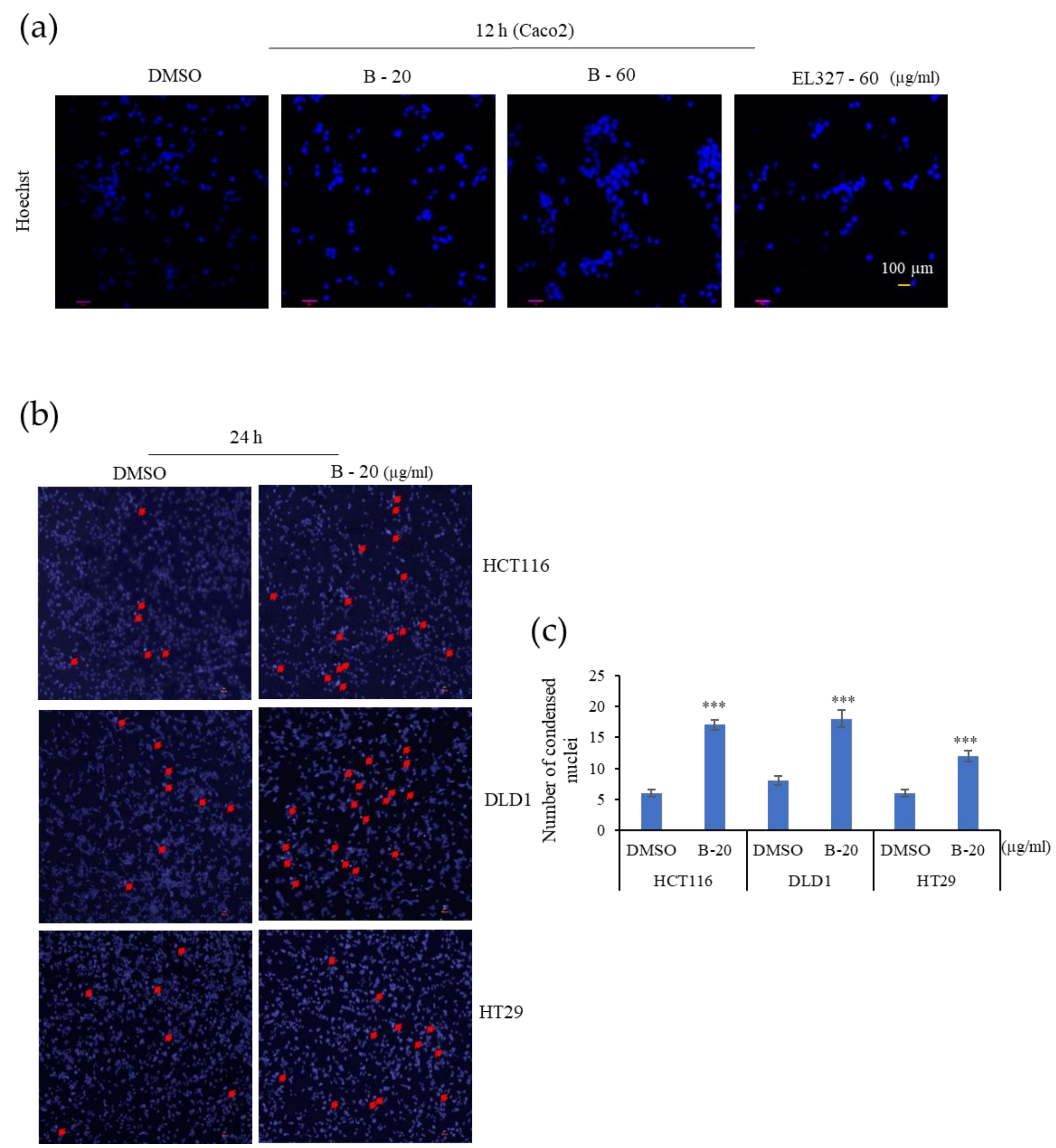

Figure S2. B induces Nuclei condensation in CRC cells. (a) Nuclei condensation of Caco2 cells upon treatment with **B** at the concentration of 20, 60 µg/ml and EL000327 at 60 µg/ml for 12 h, as determined by Hoechst staining. (b) HCT116, DLD1 and HT29 cells upon treatment with **B** at the concentration of 20 µg/ml for 24 h, as determined by Hoechst staining. Arrowheads indicate nuclear condensation in cells. (c) Quantification of condensed nuclei in HCT116, DLD1 and HT29 cells treated with 20 µg/ml of **B** for 24 h. n=3. Data represent mean ± S.D. *** $p < 0.001$, compared with the DMSO-treated control group.

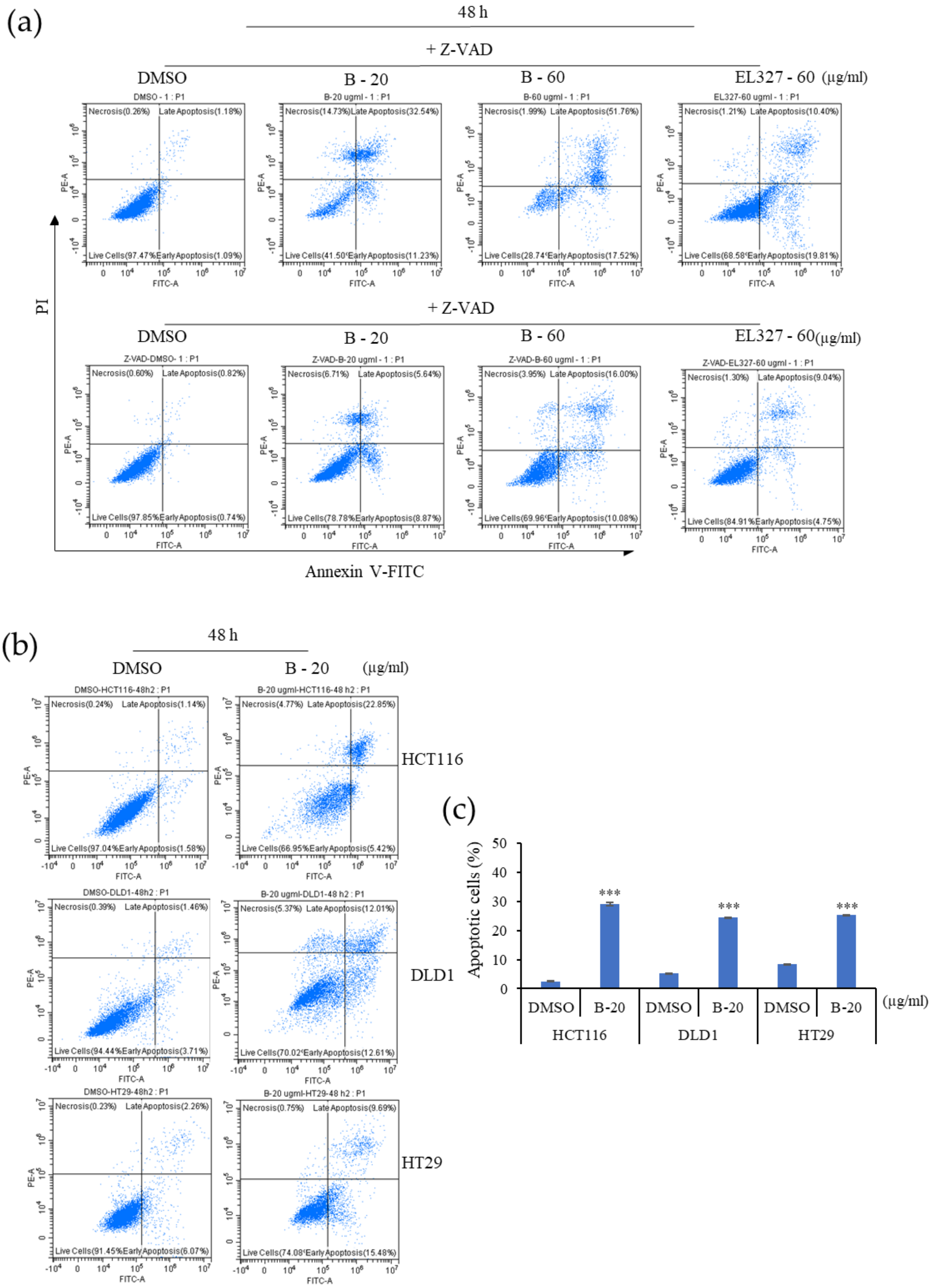

Figure S3. B induces apoptosis in CRC cells. (a) Flow cytometric analysis of dead cells stained by Annexin v-FITC (apoptotic cells) and PI (necrotic cells) upon treatment of **B** (20, 60 μg/ml) or EL000327 (60 μg/ml) for 48 h in presence or absence of Z-VAD-FMK (10 μM). (b) Flow cytometric analysis of apoptosis of HCT116, DLD1 and HT29 cells upon treatment of 20 μg/ml of **B** for 48 h. (c) Quantification of the percentage of apoptotic cells treated with 20 μg/ml of **B** and analyzed by flow cytometry. n=3. Data represent mean ± S.D. *** $p < 0.001$, compared with the DMSO-treated control group.

(a)

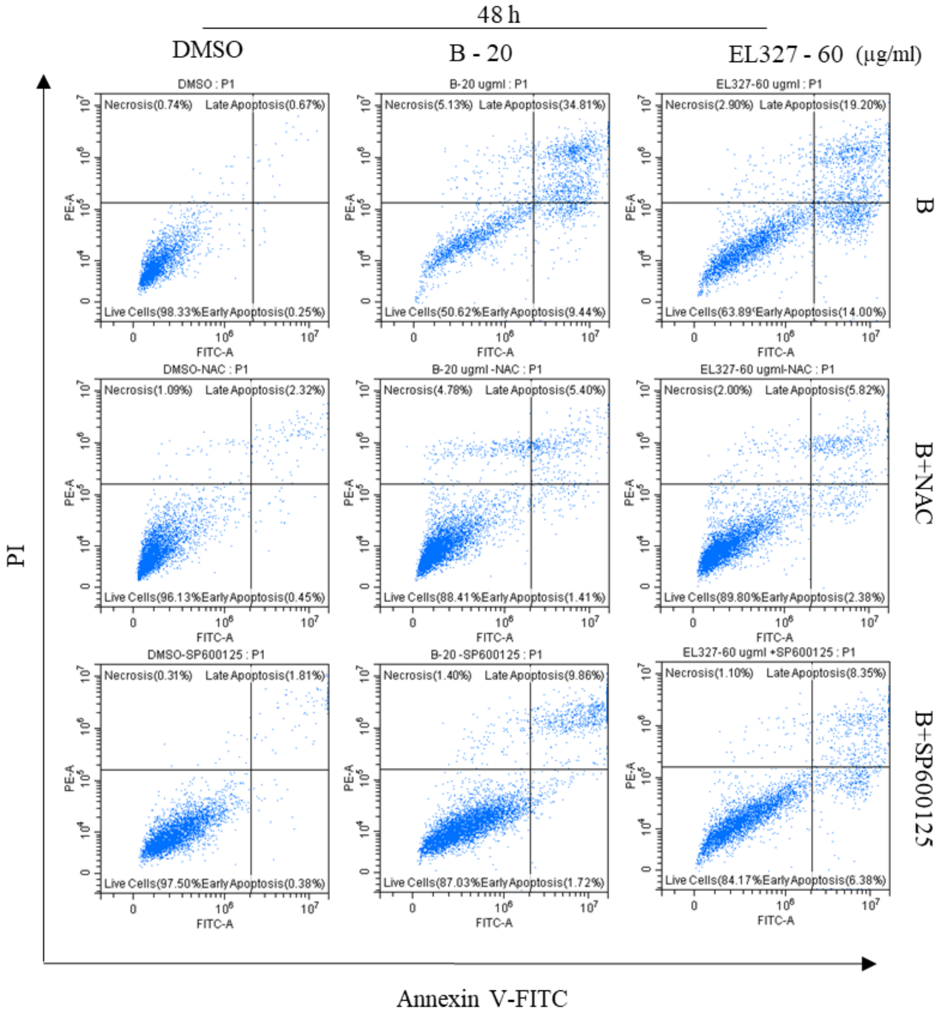

Figure S4. B decreased apoptosis in Caco2 cells in presence of ROS and JNK inhibitors. **(a)** Flow cytometric analysis of apoptotic cells stained by Annexin V-FITC and PI after treatment with **B** (20 µg/ml) or EL000327 (60 µg/ml) for 48 h, with or without NAC (5 mM) or SP600125 (10 µM).

(a)

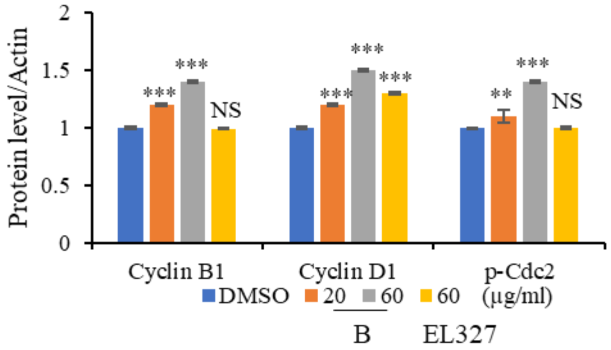

(b)

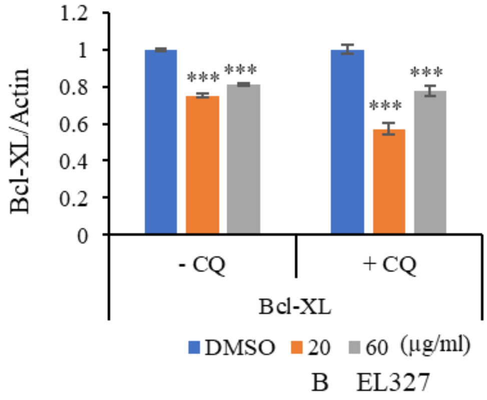

Figure S5. B modulated Cell cycle related protein expressions and anti-apoptotic protein Bcl-XL (a) Quantification of Cyclin B1, Cyclin D1 and p-Cdc2 protein expressions. (b) Quantification of Bcl-XL protein expressions in presence or absence of CQ. Data represent mean ± S.D. ** $p < 0.01$, *** $p < 0.001$; NS: no significant difference ($p > 0.05$) compared with the DMSO-treated control.

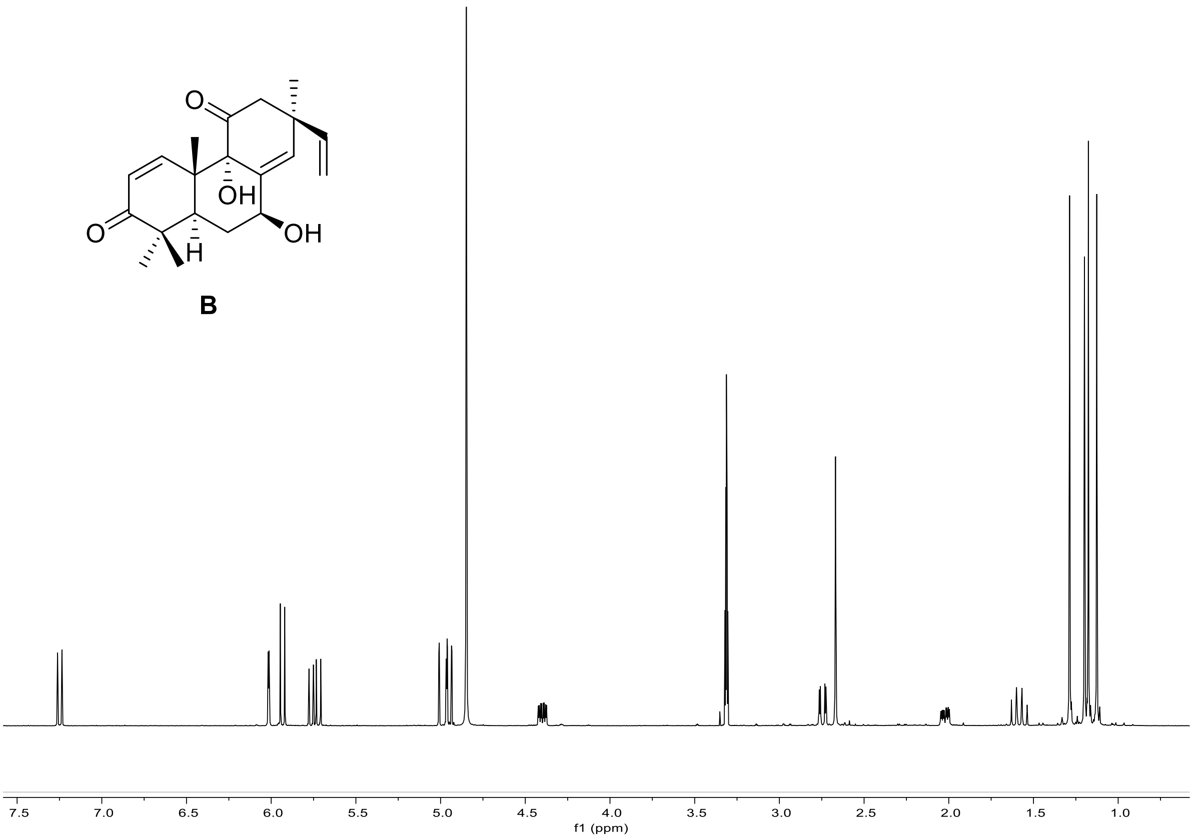

Figure S6. ^{1}H NMR spectrum of Libertellenone T (**B)** in MeOD

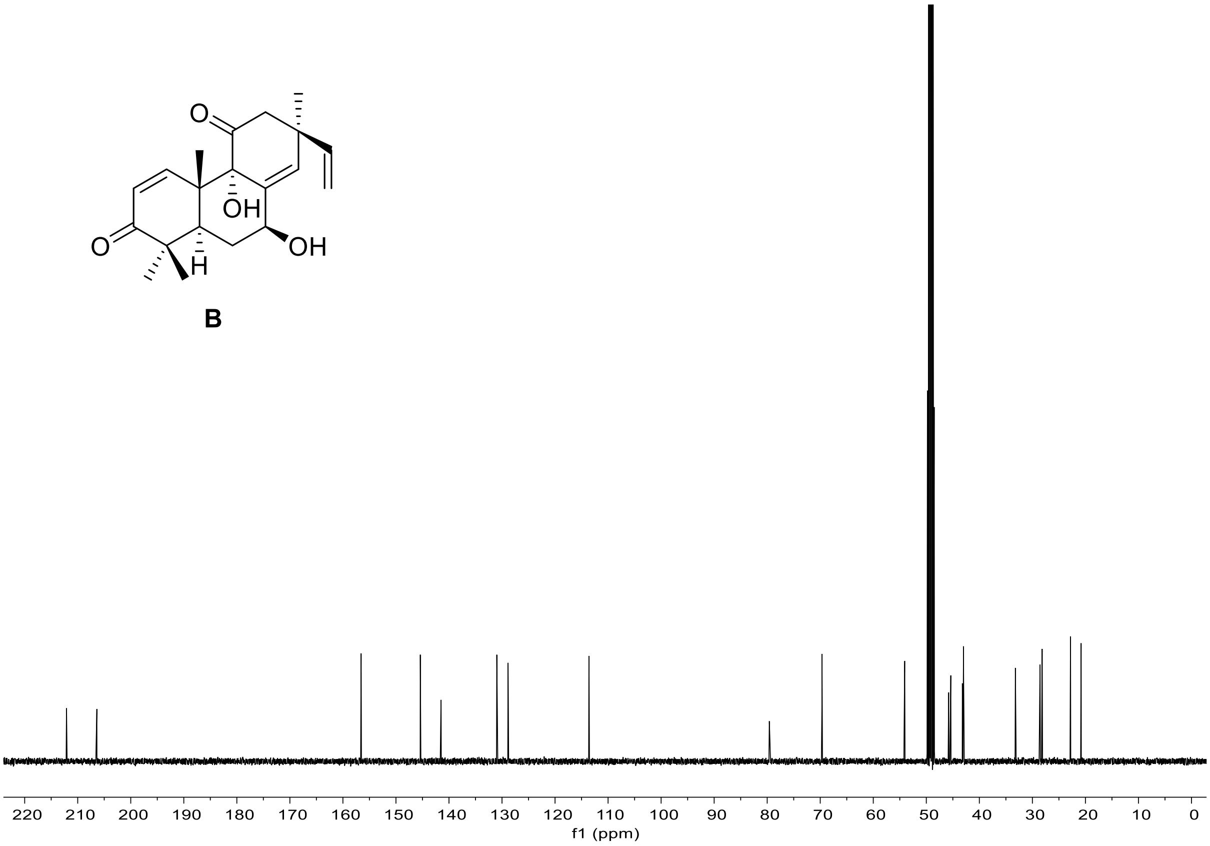

Figure S7. ^{13}C NMR spectrum of Libertellenone T (**B)** in MeOD

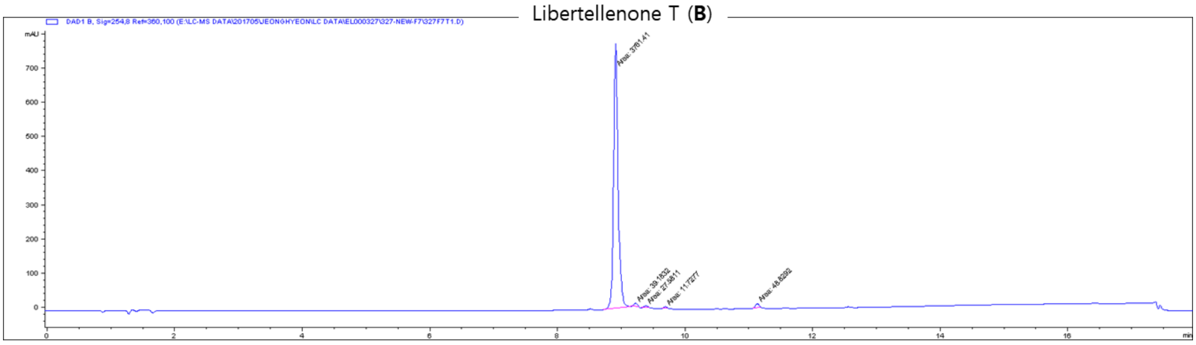

#	Time	Area	Height	Width	Area%	Symmetry
1	8.907	3761.4	777.8	0.0806	96.726	0.808
2	9.222	39.2	9.6	0.068	1.008	0.978
3	9.382	27.6	6.1	0.0748	0.709	1.044
4	9.684	11.7	4.1	0.0478	0.302	1.093
5	11.129	48.8	12.3	0.0662	1.256	0.91

Figure S8. Percentage purity of Libertellenone T (**B**)

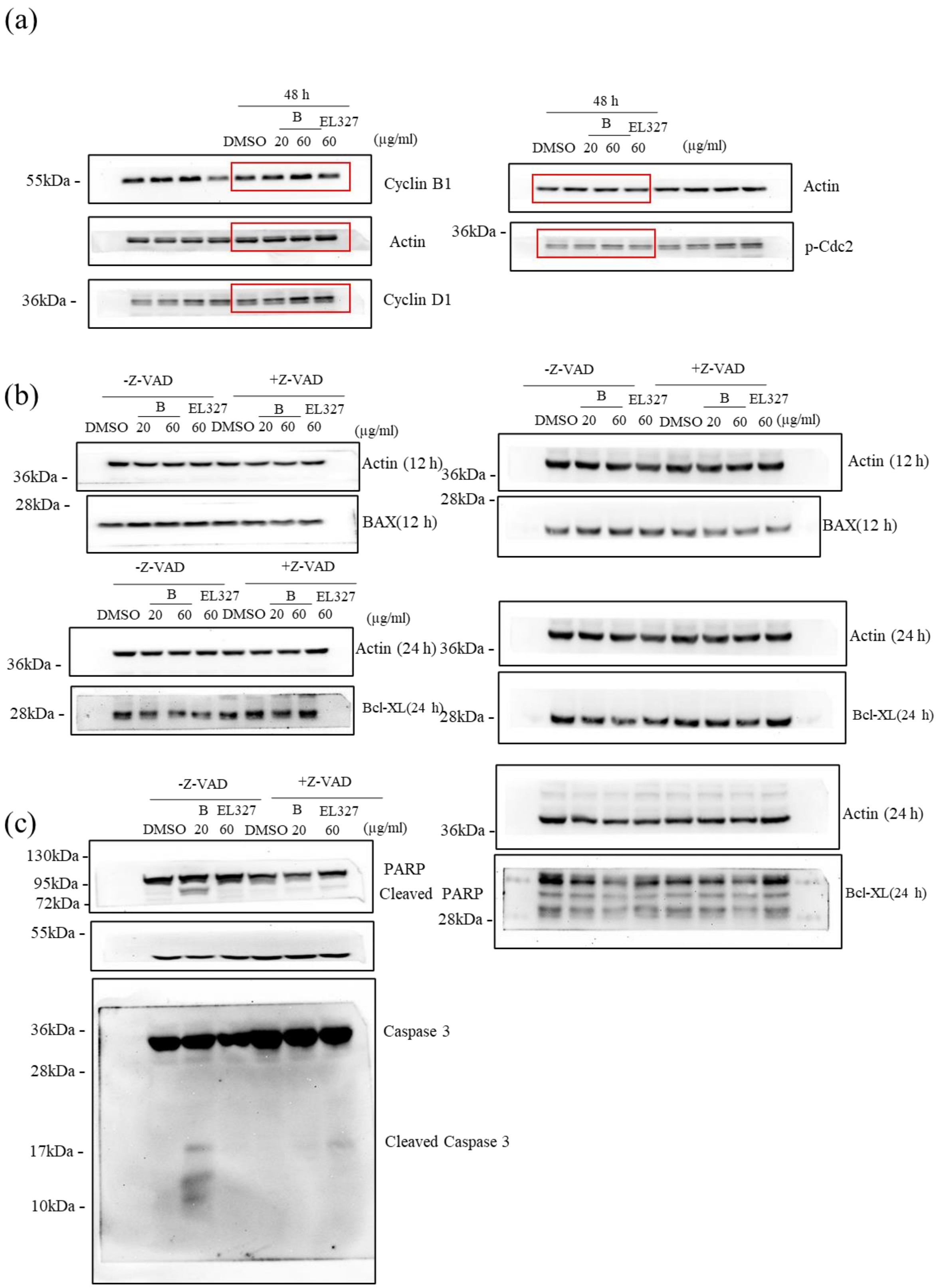
(a)
48 h
B
EL327
DMSO 20 60 60
(μg/ml)
55kDa -
Cyclin B1
Actin
36kDa -
Cyclin D1
p-Cdc2
(b)
-Z-VAD
+Z-VAD
DMSO 20 60 60 DMSO 20 60 60
Actin (12 h)
BAX(12 h)
28kDa -
Actin (24 h)
Bcl-XL(24 h)
(c)
130kDa -
95kDa -
72kDa -
PARP
Cleaved PARP
17kDa -
10kDa -
Caspase 3
Cleaved Caspase 3

(d)

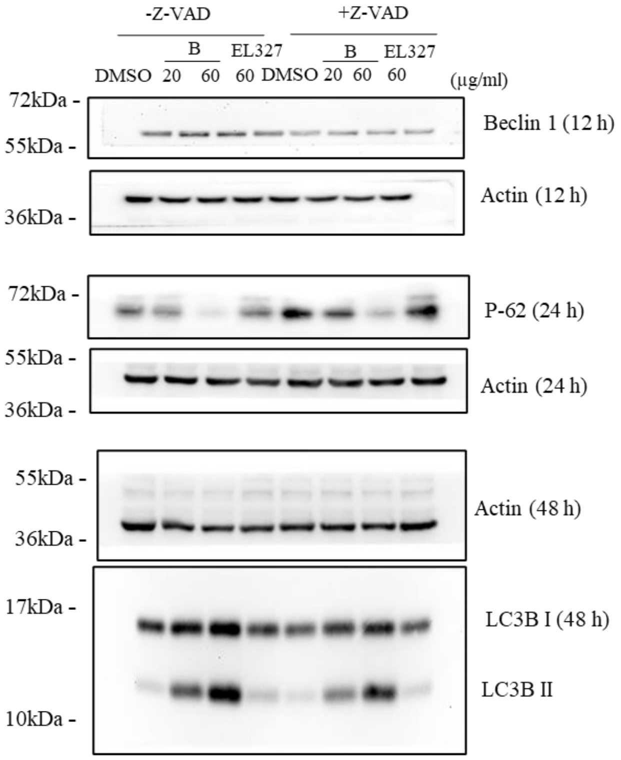
-Z-VAD
+Z-VAD
B
EL327
B
EL327
DMSO 20 60 60 DMSO 20 60 60
(μg/ml)
72kDa -
55kDa -
Beclin 1 (12 h)
36kDa -
Actin (12 h)
72kDa -
P-62 (24 h)
55kDa -
36kDa -
Actin (24 h)
55kDa -
36kDa -
Actin (48 h)
17kDa -
LC3B I (48 h)
LC3B II
10kDa -

(e)

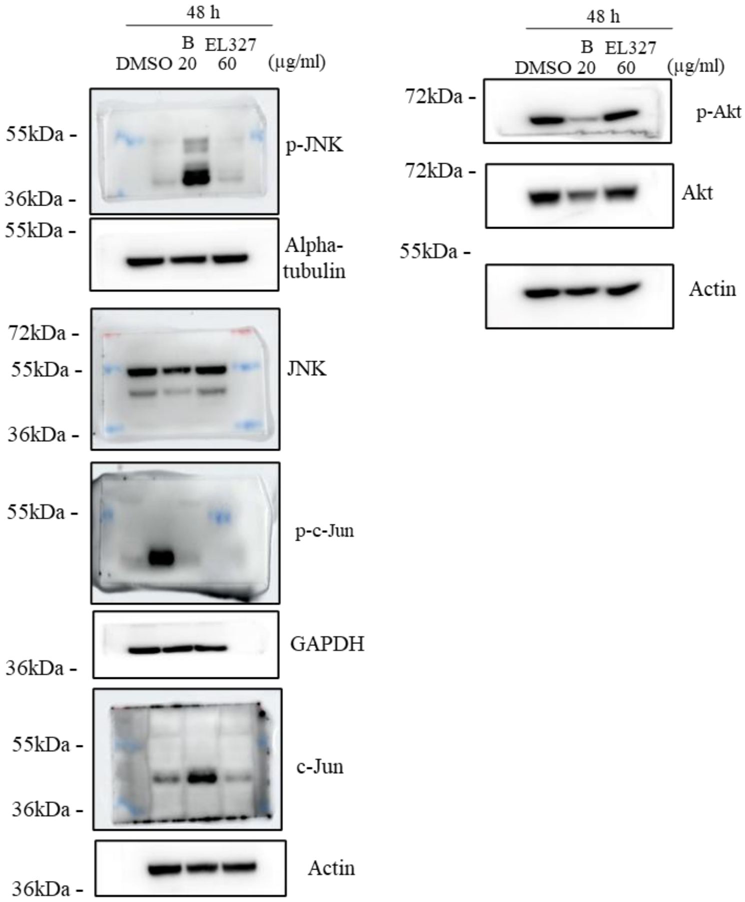
48 h
B EL327
DMSO 20 60 (μg/ml)
55kDa -
p-JNK
36kDa -
55kDa -
Alpha-
tubulin
72kDa -
55kDa -
JNK
36kDa -
55kDa -
p-c-Jun
GAPDH
36kDa -
55kDa -
c-Jun
36kDa -
Actin
36kDa -
48 h
B EL327
DMSO 20 60 (μg/ml)
72kDa -
p-Akt
72kDa -
Akt
55kDa -
Actin

(f)

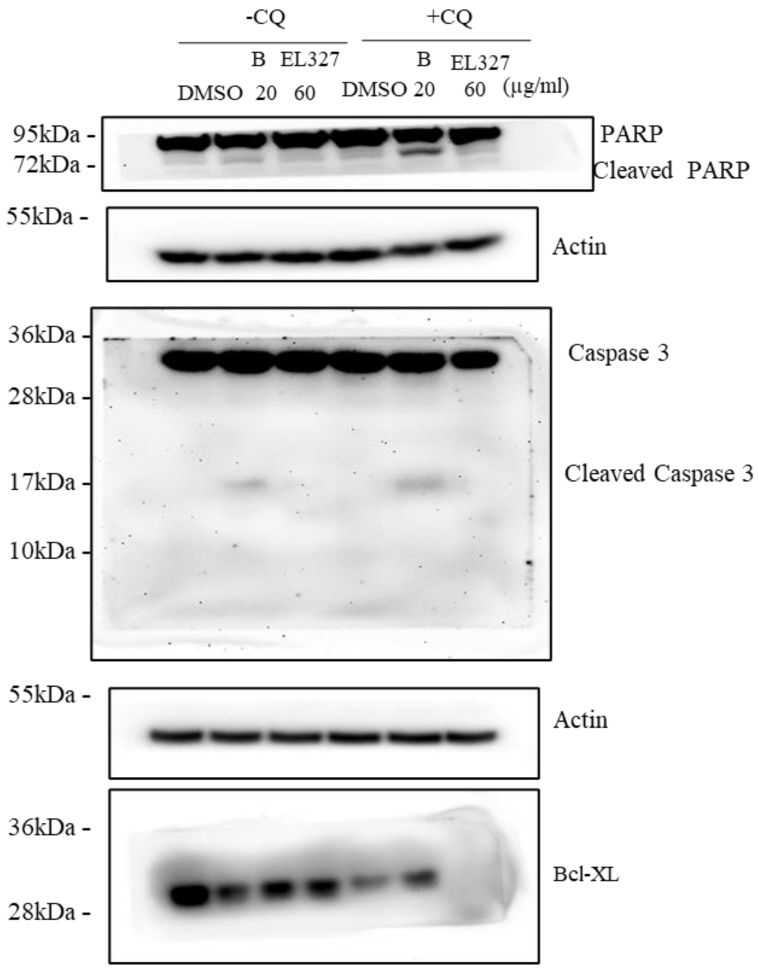
-CQ
+CQ
B
EL327
B
EL327
DMSO
20
60
DMSO
20
60
(μg/ml)
95kDa
72kDa
PARP
Cleaved PARP
55kDa
Actin
36kDa
28kDa
17kDa
10kDa
Caspase 3
Cleaved Caspase 3
55kDa
Actin
36kDa
28kDa
Bcl-XL

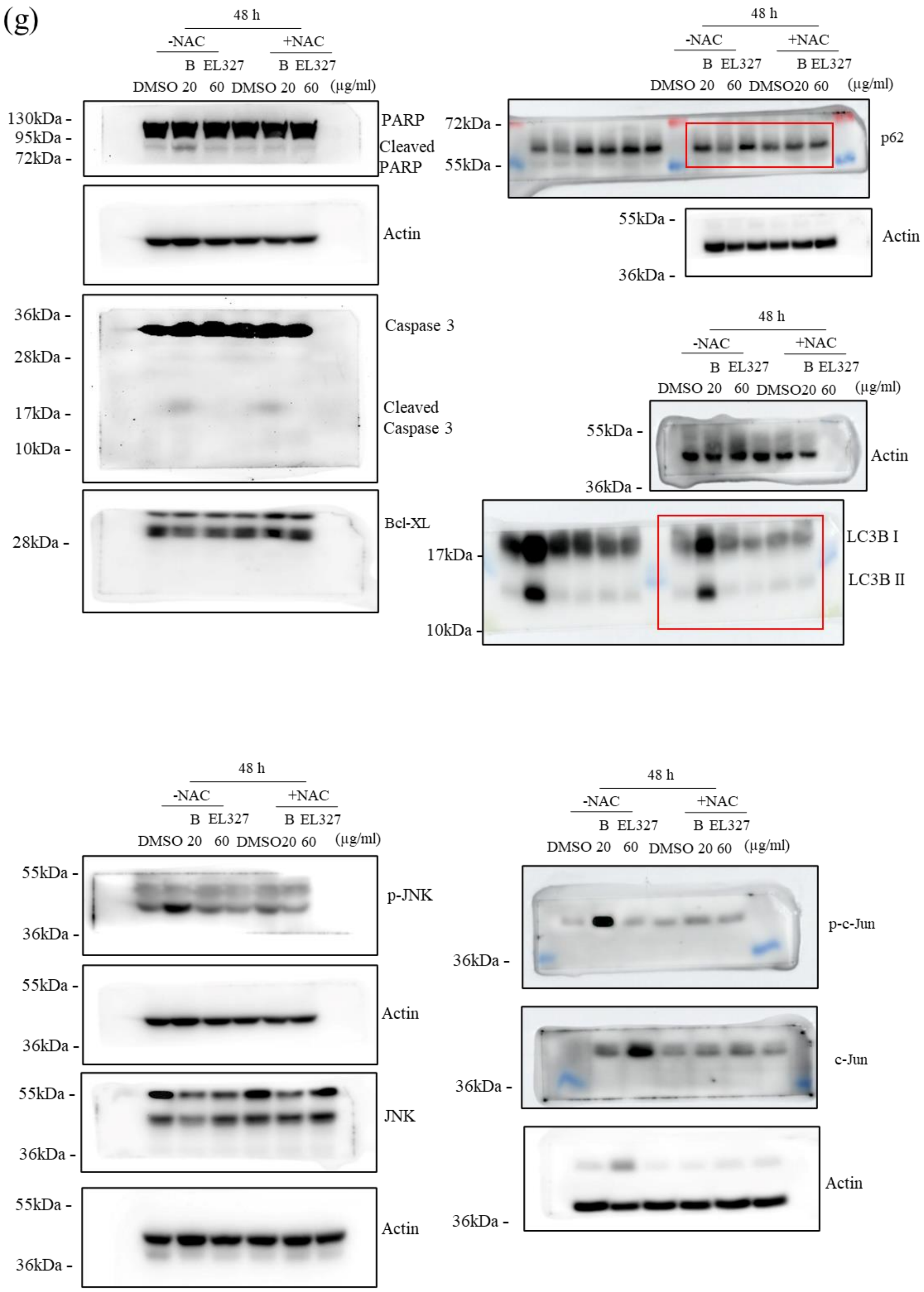

(g)
48 h
-NAC
+NAC
B EL327
DMSO 20 60 DMSO 20 60 (µg/ml)
130kDa -
95kDa -
72kDa -
PARP
Cleaved PARP
Actin
36kDa -
28kDa -
17kDa -
10kDa -
Caspase 3
Cleaved Caspase 3
28kDa -
Bcl-XL
72kDa -
55kDa -
p62
55kDa -
36kDa -
Actin
55kDa -
36kDa -
Actin
17kDa -
10kDa -
LC3B I
LC3B II
55kDa -
36kDa -
p-JNK
Actin
JNK
Actin
36kDa -
p-c-Jun
c-Jun
Actin

(h)

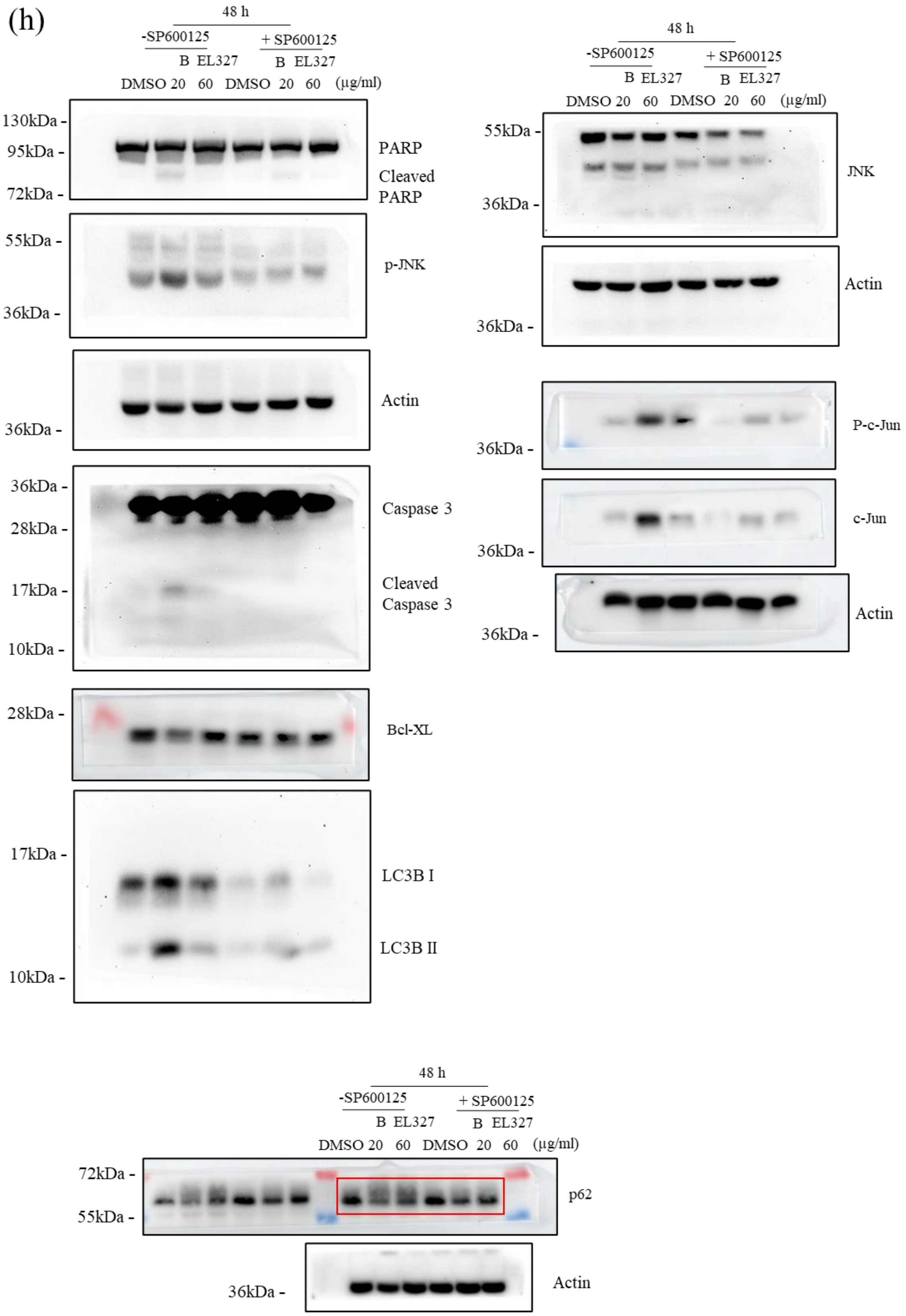
48 h
-SP600125
+ SP600125
B EL327
DMSO 20 60 DMSO 20 60 (µg/ml)
130kDa -
95kDa -
72kDa -
PARP
Cleaved PARP
55kDa -
36kDa -
p-JNK
Actin
36kDa -
28kDa -
17kDa -
10kDa -
Caspase 3
Cleaved Caspase 3
28kDa -
Bcl-XL
17kDa -
10kDa -
LC3B I
LC3B II
JNK
Actin
P-c-Jun
c-Jun
Actin
72kDa -
55kDa -
p62
36kDa -
Actin

(I)

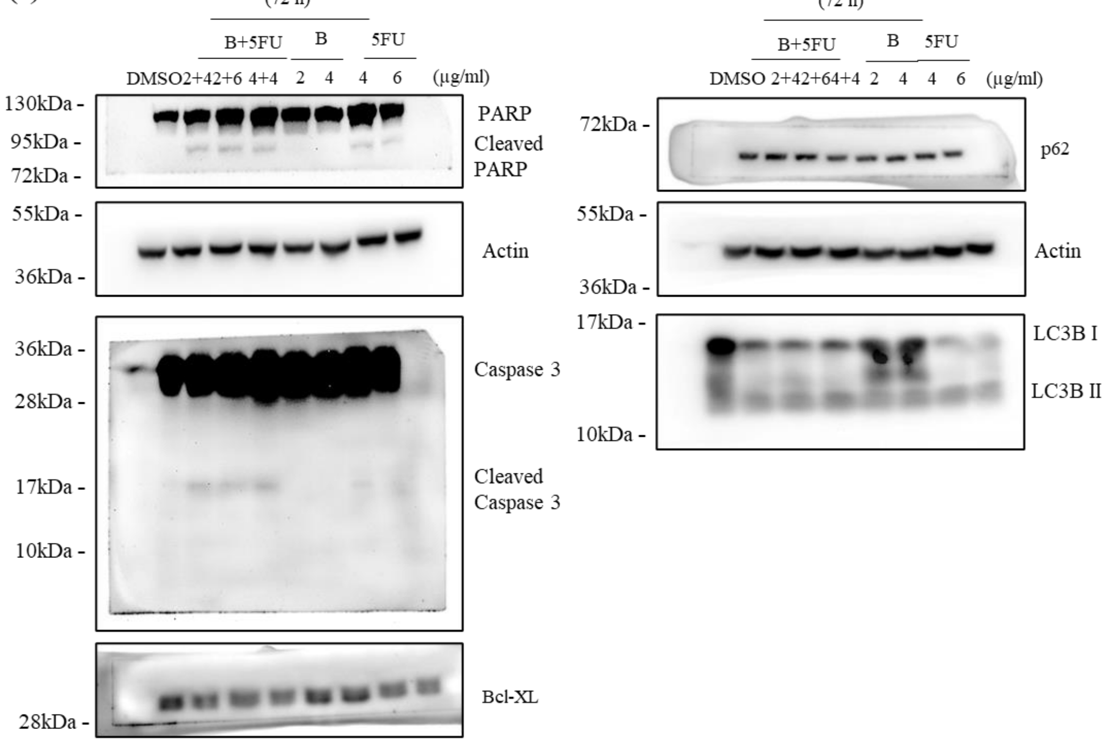

(J)

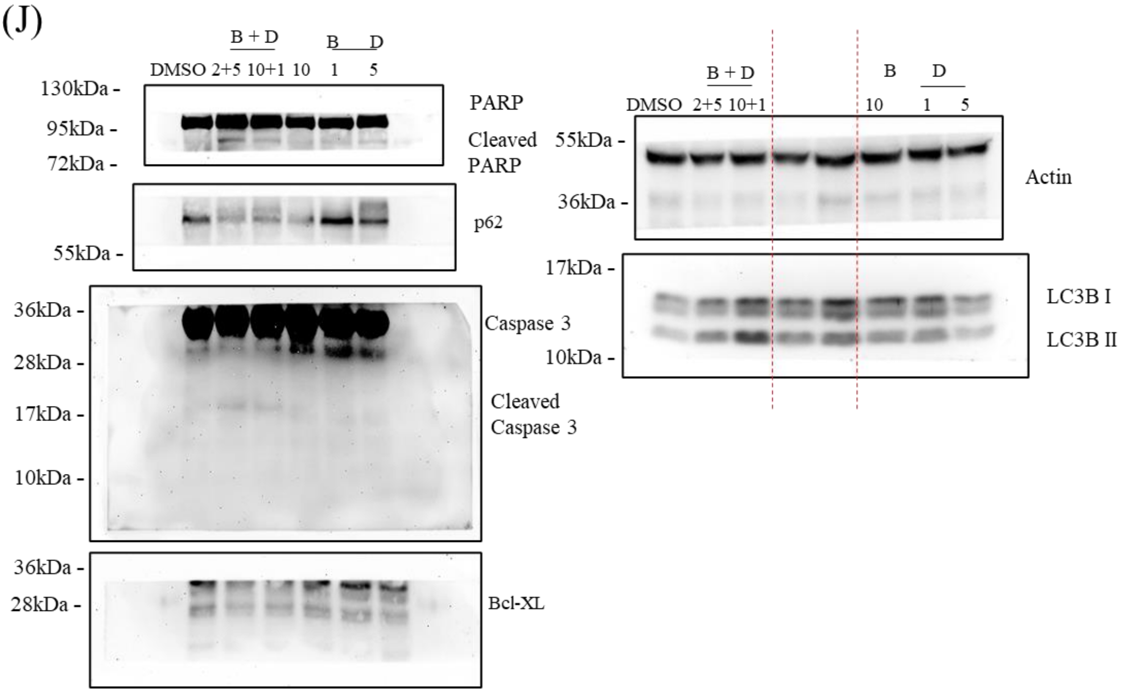

Figure S9. The raw data of western blotting
